# Unveiling the potential applications of buds of *Lonicera japonica* Thunb. var. *chinensis* (Wats.) Bak based on *in vitro* biological activities, bio-active components, and potential applications coupled to targeted metabolomics

**DOI:** 10.3389/fpls.2024.1418957

**Published:** 2024-09-26

**Authors:** Zhenying Liu, Yunxia Cheng, Yaoting Xiang, Zhimao Chao

**Affiliations:** ^1^ Institute of Chinese Materia Medica, China Academy of Chinese Medical Sciences, Beijing, China; ^2^ Graduate School of China Academy of Chinese Medical Science, Beijing, China

**Keywords:** red honeysuckle, biological activities, bio-active components, metabolomics, sensory flavor

## Abstract

**Introduction:**

The buds of *Lonicera japonica* Thunb. var. *chinensis* (Wats.) Bak, commonly named red honeysuckle, have attracted attention because of their bright colors. However, owing to the lack of systematic studies, the potential applications of red honeysuckle are not clear, and its development and utilization have not been well known.

**Methods:**

In this study, compared with the buds of *L. japonica* Thunb. (honeysuckle), the potential applications of red honeysuckle were explored based on biological activities, bio-active components, and sensory flavor combined with widely targeted metabolomics.

**Results:**

As a result, *in vitro* tests showed that it had a stronger antioxidant and a stronger inhibitory effect on the growth of *Escherichia coli* and *Staphylococcus aureus*. There was no cytotoxicity on LPS-induced RAW264.7 cells in its aqueous extract using the CCK-8 method. Moreover, it also had a stronger effect on inhibiting the expression of inflammatory factors such as interleukin-6 (IL-6), tumor necrosis factor-α (TNF-α), and interleukin-1β (IL-1β). The content of its bio-active components chlorogenic acid and cynaroside was significantly higher (*p* ≤ 0.001) than that of green honeysuckle. Widely targeted metabolomics analysis revealed that 4 volatile metabolites, such as (*E*)-4-hexene-1-ol and pyrazole, and 21 non-volatile metabolites, such as macranthoside B and oleanolic acid-3-*O*-glc(1-2)-(ara)-28-*O*-glucoside ester, were specific in red honeysuckle. Interestingly, 14 specific terpenoid metabolites were triterpenoid saponins, indicating a stronger biological activity in red honeysuckle. The sensory flavor analysis showed that the red honeysuckle had a stronger herbal and lighter floral flavor.

**Discussion:**

In conclusion, red honeysuckle had great development value with potential applications in medicines, foods, beverages, pigment additives, and health products.

## 1 Introduction

The bud of *Lonicera japonica* Thunb. (Caprifoliaceae), named green honeysuckle, is known for its medicinal and edible properties. Many studies demonstrated that it has a series of biological (antioxidant, antibacterial, and anti−inflammatory) activities (Wang et al., 2021; Mu et al., 2022). It has gained great popularity with the increase of people’s awareness of its healthcare applications. For example, it could be made into functional foods and dietary supplements to prevent chronic diseases such as arthritis, diabetes mellitus, and cardiopathy (Kirina et al., 2021; Feng et al., 2022). It could be made into herbal teas, beverages, juices, and wines because of its strong fragrance and miraculous healing effect, especially in summer (Belyaeva et al., 2021; Fang et al., 2022).

In recent years, mutants of the *Lonicerae* plants have been gradually developed and are favored by consumers (Carrillo-Galván et al., 2020; Eltaib and Alzain, 2023). Among them, *L. japonica* Thunb. var. *chinensis* (Wats.) Bak is a representative variety that has been cultivated in large areas. Its bud, named red honeysuckle, has been getting more and more attention because of its bright colors and strong fragrance. For example, Yu made a preliminary study on the red honeysuckle anthocyanin and formation mechanism (Yu, 2013). Tan et al. explored the role of anthocyanin synthase coding genes in the synthesis of red honeysuckle anthocyanins (Tan et al., 2022). Li et al. studied the breeding system and pollination biology of red honeysuckle (Li et al., 2019). Li et al. determined the optimal harvesting time of red honeysuckle by measuring the flowers’ thousand bud weight of different flowering phase (Li et al., 2013). Yuan et al. compared the anthocyanin content of red and traditional green honeysuckle (Yuan et al., 2014). Hu et al. determined the active compounds in different organs of red honeysuckle cultivated in saline soil by high-performance liquid chromatography (HPLC) (Hu et al., 2017). However, its biological activities and chemical composition have been less studied, resulting in its potential development value remaining unclear. Therefore, systematic studies on the biological activities and chemical composition of red honeysuckle are necessary for its development and commercialization as a new food raw material.

In this study, in order to comprehensively explore the potential applications of red honeysuckle and provide data support for the development of related products, its biological activities (such as antioxidant, antibacterial, and anti-inflammatory), bio-active components (such as chlorogenic acid, cynaroside, and rutin), metabolites (such as volatile and non-volatile metabolites), and sensory flavor were analyzed and compared with those of green honeysuckle.

## 2 Materials and methods

### 2.1 Chemicals and reagents

6-Hydroxy-2,5,7,8-tetramethylchroman-2-carboxylic acid (Trolox), ferric chloride hexahydrate, 2,2′-azino-bis (3-ethylbenzothiazoline-6-sulfonic acid) diammonium salt (ABTS), Folin–Ciocalteu phenol, and 1,1-diphenyl-2-picrylhydrazyl (DPPH) were obtained from Sigma−Aldrich (St. Louis, MO, USA). All standards (purities ≥ 98%) of the HPLC method were purchased from Push Bio−Technology Co., Ltd. (Chengdu, China). The internal standard (*p*-xylene-d10) of the gas chromatography–mass spectrometry (GC-MS) method was purchased from Yien Chemical Technology Co., Ltd. (Shanghai, China). HPLC- and UPLC-grade solvents were from Fisher Scientific (Waltham, MA, USA). Analytical-grade solvents were purchased from Fuyu Fine Chemical Co., Ltd. (Tianjin, China). The mouse interleukin-6 (IL-6) (SEKM-0007), interleukin-1β (IL-1β) (SEKM-0002), tumor necrosis factor-α (TNF-α) (SEKM-0034) ELISA kit, and PBS buffer were purchased from Solarbio Science & Technology Co., Ltd. (Beijing, China). The anthocyanin content detection kit was from Zike Biological Technology Co., Ltd (Shenzhen, China). Dexamethasone, fetal bovine serum (FBS), lipopolysaccharides (LPS), and Griess reagent were purchased from Sigma−Aldrich Co., Ltd. DMEM basic (1×) and PBS pH 7.4 basic (1×) were from ThermoFisher Biochemical Products (Beijing) Co., Ltd. The Cell Counting Kit-8 (CCK-8, Biosharp) was from Beijing Labgic Technology Co., Ltd. Penicillin–streptomycin solution was manufactured by HyClone Laboratories.

### 2.2 Bacterial strains and culture media

All bacterial strains, including *Escherichia coli* (ATCC 25922), *Staphylococcus aureus* (ATCC 29213), and *Bacillus subtilis* (ATCC 6633), were acquired from Guangdong Microbial Culture Collection Center (Guangzhou, China).

LB broth culture medium and agar were obtained from Sangon Biotech (Shanghai) Co., Ltd. (Shanghai, China).

### 2.3 Sample preparation

**Figure 1:**
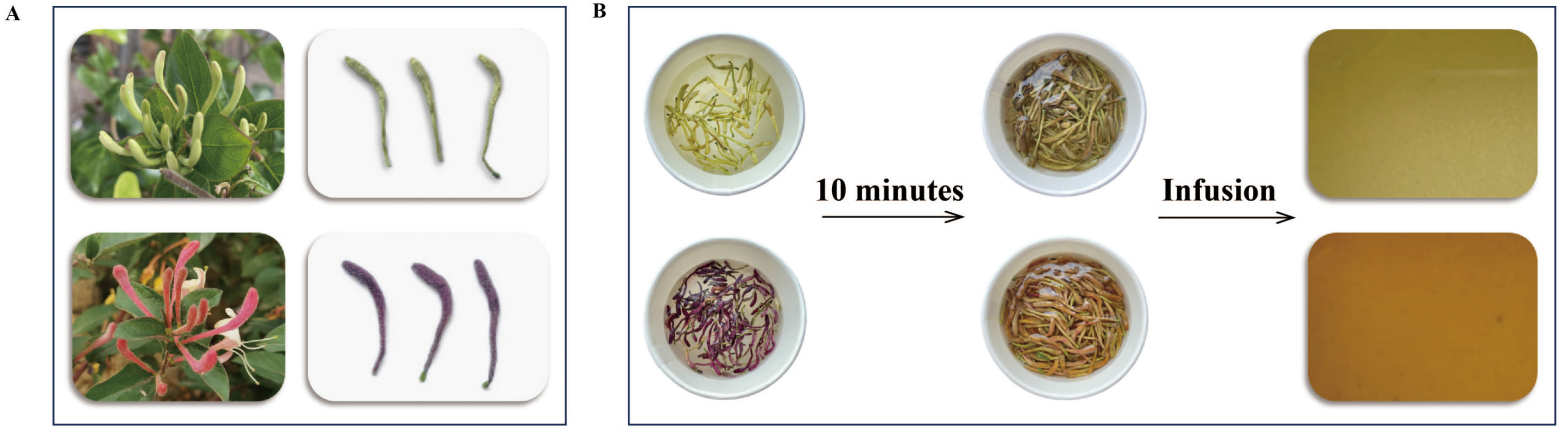
The appearance **(A)** and tea infusion **(B)** of green and red honeysuckles.

As shown in **Figure 1**, the buds of *L. japonica* Thunb. (green honeysuckle) were collected from a planting base located in Julu County of Hebei Province, and the buds of *L. japonica* Thunb. var. *chinensis* (Wats.) Bak (red honeysuckle) were obtained from a planting base located in Henan Academy of Agricultural Sciences. The buds were collected in the morning of flowering and then dried so that their moisture content met the requirements of the Chinese Pharmacopoeia. Then, they were crushed into powder (particle size: 40 mesh) by a high-speed crusher and placed in a dryer for subsequent use.

### 2.4 *In vitro* antioxidant activity determination

The *in vitro* antioxidant activities were determined by three assays based on a previous study (Liu et al., 2023), namely, ABTS and DPPH radical scavenging activity and ferric reducing antioxidant power (FRAP). Correspondingly, measurement of the absorbance of the solution was performed at 734, 517, and 593 nm, respectively. Their results were calculated by a calibration curve and expressed as μTrolox equivalent per gram (TE μ/g).

### 2.5 *In vitro* antibacterial activity determination

The *in vitro* antibacterial activity of the aqueous extracts was estimated against three bacteria strains listed above using the agar plate-counting technique.

#### 2.5.1 Cell suspension preparation

LB liquid medium (3 mL) was added to four bacterial culture tubes. Single colonies of the above three bacteria strains were selected and transformed to the liquid medium, and the other one was chosen as a blank control. Then, they were incubated for 15 h in an oscillator (37°C, 200 rpm).

#### 2.5.2 Aqueous solution extraction

Samples (30 g) and 300 mL of water were added to a casserole, soaked for 40 min, and boiled for approximately 30 min. Then, the mixture was filtered with gauze. Next, the filtrate was poured out and repeatedly decocted. After the two filtrates were combined, they were freeze-dried to obtain aqueous extracts. Then, the extracts were placed in centrifuge tubes and placed under ultraviolet lamps for UV sterilization for more than 30 min for later use.

#### 2.5.3 Plate coating count

Firstly, sterile PBS solution was added to dilute the bacterial solution to 1×10^6^ colony-forming units (CFU)/mL. Next, 2 mL of diluted bacterial solution was added into the corresponding centrifuge tubes, while the extracts were not added in the blank group. They were then incubated at 37°C for 24 h. Similarly, the bacterial solution was diluted 10 times. After uniformly coating LB solid medium with 100 μL of diluent, 18 h of incubation at 37°C was performed. Finally, the colonies were photographed and counted, and the antibacterial rate was calculated by the following formula:


Bacterial concentration (bc)=colony number×dilution factor×10

$$\text{Antibacterial rate }\left(\%\right)=[1-{\left(\text{experimental}/\text{control}\right)}_{\text{bc}}\times 100\%]$$

### 2.6 *In vitro* anti-inflammatory activity determination

#### 2.6.1 Cell culture and viability

CCK-8 was used to detect the cell viability of two aqueous extracts of honeysuckle at different concentrations in RAW264.7 cells. In detail, RAW264.7 cells were cultured in DMEM (Dulbecco’s minimal essential medium) containing 10% FBS (fetal bovine serum) and 1% double antibiotic in an incubator with 37°C and 5% CO_2_ for 24 h. Then, the different concentrations of two aqueous extracts (1,000, 800, 400, 200, 100, 50, 25, 12.5, and 0 μg/mL) were added for the next 24 h. Subsequently, CCK-8 (10 μL/well) was added and incubated for 2 h. After shaking, the absorbance of each well was measured at 450 nm and cell viability was calculated.


$$Cell\;viability\;\%=\left({\text{A}}_{S}-{\text{A}}_{B}\right)/\left({\text{A}}_{C}-{\text{A}}_{B}\right)$$

where *A*_S_ indicates the absorbance of the sample, *A*_B_ indicates the absorbance of the blank, and *A*_C_ indicates the absorbance of the control.

#### 2.6.2 NO production by Griess

The NO inhibitory effects of two aqueous extracts in LPS-induced RAW264.7 cells were detected by Griess assay. Specifically, the control group, model group (LPS), DXMS group (dexamethasone), and sample groups (400, 800, and 1,000 μg/mL of two aqueous extracts were named G-L, G-M, G-H, R-L, R-M, and R-H, respectively) were set up, respectively. The RAW264.7 cells (5×10^4^ cells/well) were cultured for 24 h. Next, according to the setting, DMEM and drug solutions were given, respectively. Then, 2 h later, LPS (last concentration was 1 μg/mL) was added to the model and administration groups and then cultured for 24 h. Finally, NO production in the supernatant solution was measured using the Griess assay.

#### 2.6.3 Inflammatory factor determination by ELISA

The experimental group was the same as that in section 2.6.2. The RAW264.7 cells (5×10^5^ cells/well) were cultured for 24 h. Then, the different concentrations of two aqueous extracts and DXMS solutions were added. After 2 h, the LPS solution (last concentration was 1 μg/mL) was added to the model and administration groups for 24-h culturing. Next, the supernatants were collected and used for ELISA to determine the concentrations of three inflammatory factors, namely, IL-6, IL-1β, and TNF-α. According to the instructions of the ELISA kit, the sample and the standard, the biotinized antibody working solution, the enzyme binding working solution, the chromogenic substrate, and the termination solution were added successively and incubated according to the requirements, respectively. Finally, the absorbance was measured at 450 nm using an enzyme-labeled instrument.

#### 2.6.4 Real time-quantitative polymerase chain reaction

The groups were the same as those in section 2.6.2. Firstly, RAW264.7 cells (1×10^6^ cells/well) were cultured for 24 h. Then, the different solutions were added according to the group setting. After 24 h, LPS (last concentration was 1 μg/mL) was added to the groups except for the control group for the next 6 h. Finally, the supernatant was removed and 500 μL of TRIzol reagent was added to extract the total RNA of cells, and the relative mRNA expressions of IL-6, IL-1β, and TNF-α were measured by real time-quantitative polymerase chain reaction (rt-qPCR).

Then, the samples were reverse-transcribed and amplified according to the reverse transcription kit SynScript^®^III RT SuperMix for qPCR. The cDNA product obtained by reverse transcription was threefold diluted and amplified as a template for rt-qPCR. The primer sequences are shown in **Supplementary Table S1**.

### 2.7 Bio-active components analysis

The sample powder was extracted with 50% methanol and analyzed using an HPLC system (Shimadzu, Kyoto, Japan). The chromatography condition was consistent with previous studies (Liu et al., 2023). Next, 10 bio-active components, namely, chlorogenic acid, cynaroside, neochlorogenic acid, caffeic acid, isochlorogenic acid A, isochlorogenic acid B, isochlorogenic acid C, cryptochlorogenic acid, lutin, and quercetin, were analyzed qualitatively and quantitatively by reference to the standard solution.

### 2.8 GC-MS analysis

#### 2.8.1 Sample preparation

The 0.5-g honeysuckle powder was weighed and transferred to a head-space vial. Then, the vial was sealed and placed at 60°C for 5 min. Next, a 120-µm divinylbenzene/carboxen/polydimethylsiloxane (DVB/CAR/PDMS) filter (Agilent, Palo Alto, CA, USA) was exposed to the head space for 15 min at 60°C. Subsequently, the samples were analyzed by the GC-MS system.

The GC-MS conditions were similar to those in another study (Wang et al., 2021), except for the heating procedure. In this study, the initial temperature setting was 40°C and was held for 3.5 min. Then, the temperature was increased to 100°C at 10°C/min, 180°C at 7°C/min, 280°C at 25°C/min, and held for 5 min.

#### 2.8.2 Qualitative and quantitative analyses

Volatile metabolites were identified using the NIST 14 standard library with a similarity of >80% based on the characteristic ion chromatogram and mass spectrum value (Li et al., 2023). Then, the concentration of each metabolite was calculated by reference to the internal standard (*p*-xylene-d10).

#### 2.8.3 Sensory flavor annotation

In order to fully compare the difference in flavor between the two kinds of honeysuckle, the flavor sensory annotation was carried out on the different metabolites selected. With reference to a series database, including the TGSCIS database (The Good Scents Company Information System, http://www.thegoodscentscompany.com), the perflavory database (http://perflavory.com/), the odour database (http://www.odour.org.uk/odour/index.html), or a related database (http://foodflavorlab.cn/#/home), combined with the comparison of the relative content of metabolites focused on the same flavor, the odor difference of the two kinds of honeysuckle was finally explored.

### 2.9 UPLC-QTRAP-MS/MS analysis

#### 2.9.1 Sample analysis

The 0.5-g honeysuckle powder was extracted with 70% methanol. Then, the extract was centrifuged and filtered for subsequent analysis.

The ExionLC™ AD UPLC system (AB, Framingham, MA, USA) coupled to an Agilent SB−C18 column (100 mm × 2.1 mm, 1.8 μm) was used to achieve chromatographic separation. The solvent system was water (A) and acetonitrile (B) with 0.1% formic acid. The gradient program was as follows: 0 min, 95% A, 0–9 min, 95%–5% A, 9–10 min, 5% A, 10–11.10 min, 5%–95% A, and 11.10–14 min, 95% A. The column temperature was 40°C. The injection volume was 2 μL. Finally, the effluent was further analyzed by electron spray ionization (ESI)−triple quadrupole−linear ion trap (QTRAP)–MS. Moreover, the MS condition was similar to the other report (Wang et al., 2021).

#### 2.9.2 Qualitative and quantitative analyses

The data were processed as described elsewhere (Zou et al., 2020). All metabolites were annotated combined with the Metware in−house MS^2^ spectral tag (MS2T) database (Wuhan Metware Biotechnology Co., Ltd., Wuhan, China) (http://www.metware.cn/) and public metabolite databases including MassBank (http://www.massbank.jp), KNAPSAcK (http://kanaya.naist.jp/KNApSAcK), human metabolome database (HMDB, http://www.hmdb.ca), MoTo DB (http://www.ab.wur.nl/moto), and METLIN (http://metlin.scripps.edu/index.php). For the quantitative analysis, the result was expressed as the mass spectrum peak area.

### 2.10 Statistical analysis

The multivariate statistical analysis was performed using the SIMCA 14.1 software (Umerics, Umeå, Sweden). All results were visualized by R (http://www.r-project.org), chiplot (https://www.chiplot.online/), and GraphPad Prism 9.0.0 (GraphPad, San Diego, CA, USA).

## 3 Results

### 3.1 *In vitro* antioxidant activity analysis

**Figure 2:**
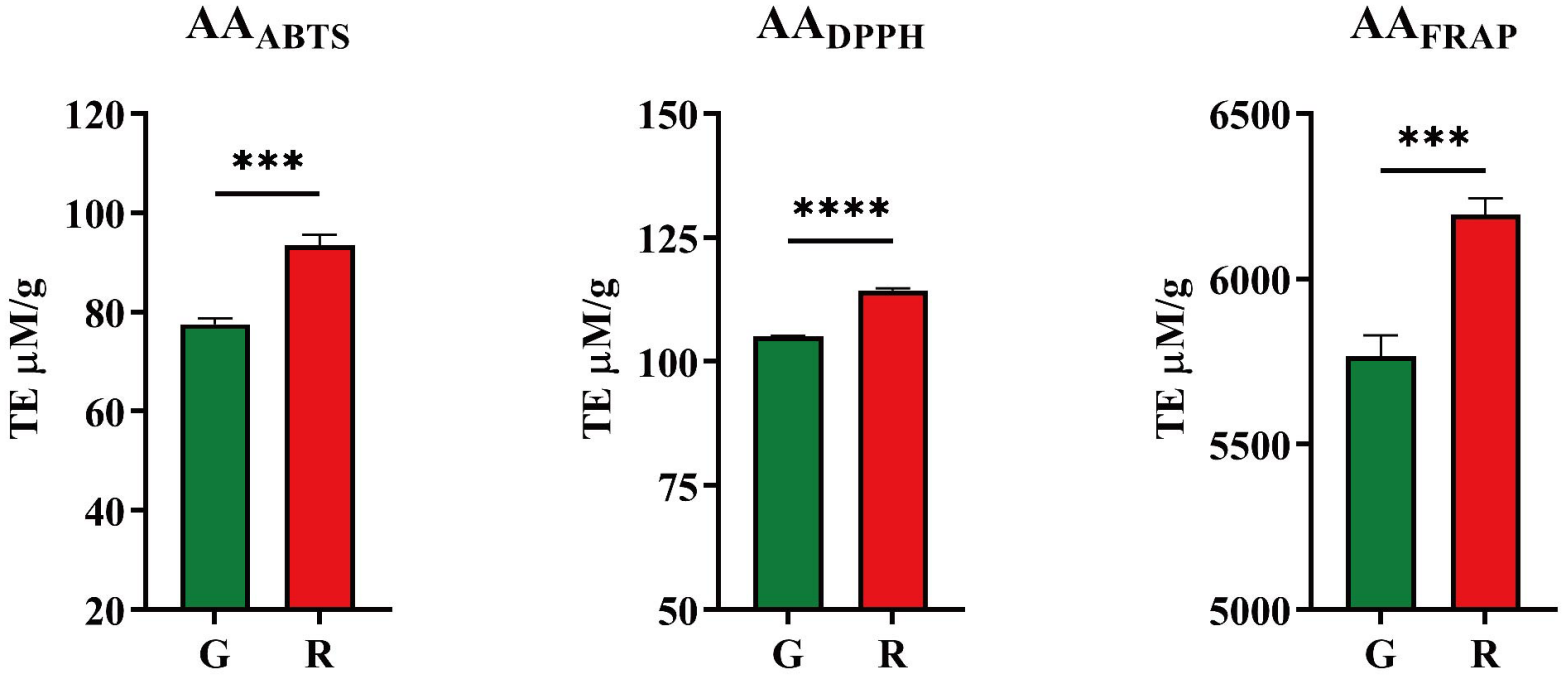
Antioxidant activities of honeysuckles. G, green honeysuckle; R, red honeysuckle. ***p ≤ 0.001, ****p ≤ 0.0001.

Three methods, including ABTS, DPPH, FRAP, based on different principles were used to evaluate the antioxidant activities due to the efficiency of antioxidants in biological systems may differ. Their results are shown in **Figure 2**.

For red honeysuckle, the average antioxidant activity evaluated by ABTS, DPPH, and FRAP was 93.44 TE μM/g, 114 TE μM/g, and 6,195 TE μM/g, respectively, while for green honeysuckle, the corresponding values were 77.58 TE μM/g, 105.1 TE μM/g, and 5,766 TE μM/g. The results of FRAP were significantly higher than those of ABTS and DPPH, which may be due to the different principles of these methods. The FRAP method aims to determine the ability of a substance to reduce iron ions, whereas ABTS and DPPH aim to determine the ability to remove corresponding free radicals.

### 3.2 *In vitro* antibacterial activity analysis

**Figure 3:**
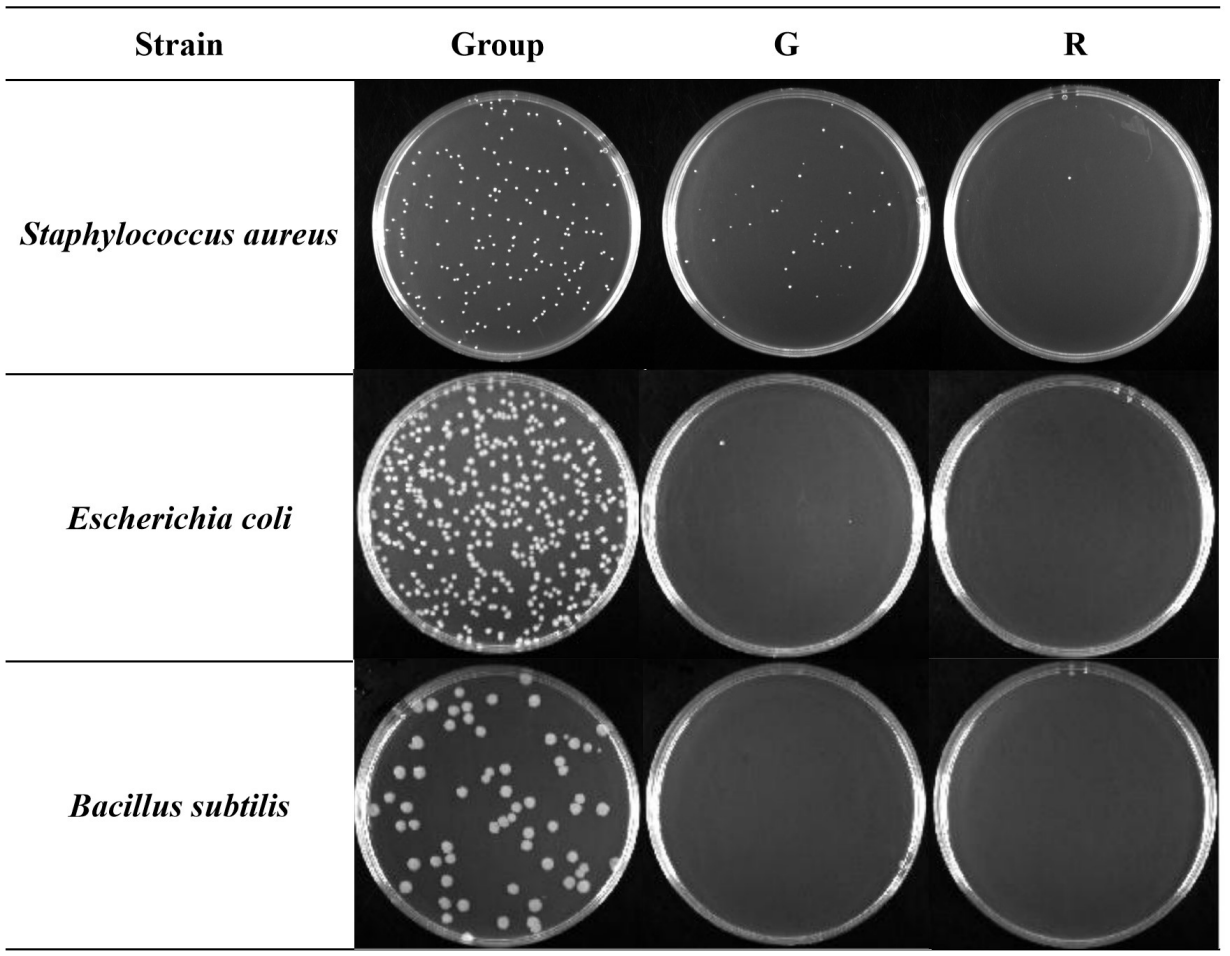
Antibacterial effect of two kinds of honeysuckle under the same dilution ratio (10^3^). G, green honeysuckle; R, red honeysuckle.

The ability of the aqueous extract of two kinds of honeysuckle to inhibit the growth of *E. coli*, *S. aureus*, and *B. subtilis* was evaluated. The average colony number and the antibacterial rate at the corresponding dilution ratio are shown in **Table 1**, and the inhibitory effect on colony growth at the same dilution ratio (10^3^) is shown in **Figure 3**.

**Table 1:** Plate counting test results (*n* = 3).

Strain	Group	Colony number	Dilution ratio	Concentration(CFU/mL)	Antibacterial rate (%)
*Staphylococcus aureus*	Control	143	10^3^	1.4 × 10^6^	/
	G	35	10^3^	3.5 × 10^5^	75.52
	R	105	10^1^	1.1 × 10^4^	99.27
*Escherichia coli*	Control	253	10^3^	2.5 × 10^6^	/
	G	33	10^2^	3.3 × 10^4^	98.69
	R	27	10^1^	2.7 × 10^3^	99.89
*Bacillus subtilis*	Control	60	10^3^	6.0×10^5^	/
	G	0	10^1^	<10^2^	100.00
	R	0	10^1^	<10^2^	100.00

The number of colonies was the counting result of each group at the corresponding dilution ratio. The counting principle was based on the national standard GB4789.2-2016, and the number of colonies was selected to be between 30 and 300 CFU for colony counting.

Compared with the control group, for *S. aureus*, the antibacterial rate of green honeysuckle was 75.52%, and that of red honeysuckle was as high as 99.27%, and there was a significant difference (*p* < 0.01) between them. For *E. coli*, the antibacterial rate of green honeysuckle was 98.69% and that of red honeysuckle was 99.89%, but there was no significant difference. For *B. subtilis*, both of them showed significant antibacterial effect, and the antibacterial rate was 100%.

This result showed that there were fewer colonies in the medium corresponding to the red honeysuckle under the same dilution ratio. *B. subtilis* had no colony growth under the inhibition of the two extracts. Combining the antibacterial rate of *S. aureus* and *E. coli*, it could be speculated that the inhibitory effects of red honeysuckle were stronger than those of green honeysuckle.

### 3.3 *In vitro* anti-inflammatory activities analysis

**Figure 4:**
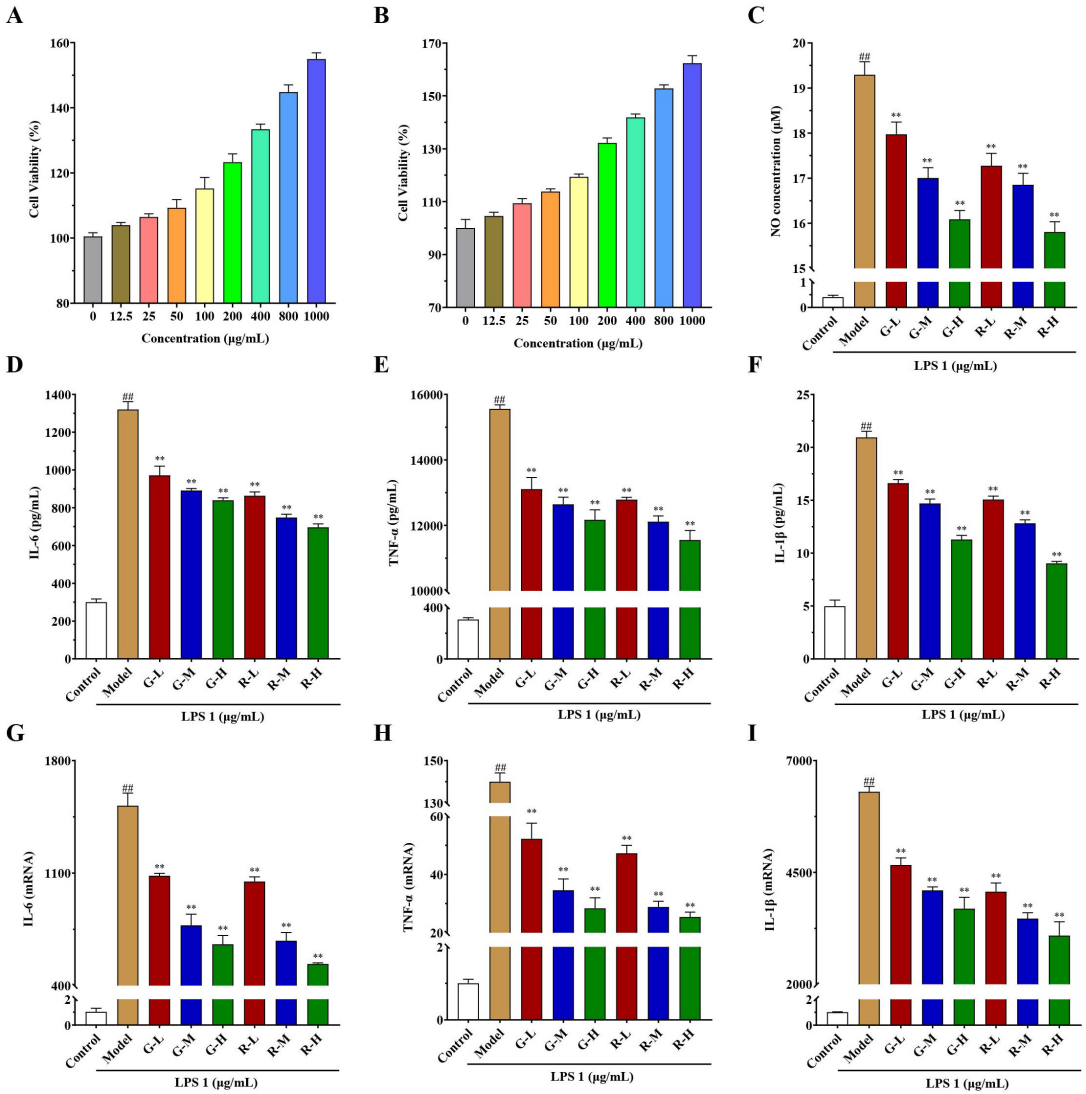
*In vitro* anti-inflammatory activity results of honeysuckle aqueous extracts on the LPS-induced RAW 264.7 cells. **(A)** Cell viability of green honeysuckle. **(B)** Cell viability of red honeysuckle. **(C)** The concentrations of NO in different groups. **(D–F)** The concentrations of inflammatory factors in the supernatant, IL-6, TNF-α, and IL-1β, respectively. **(G–I)** The relative mRNA expression of IL-6, TNF-α, and IL-1β, respectively. G-L, G-M, and G-H: the concentrations of green honeysuckle were 400, 800, and 1,000 μg/mL, respectively; R-L, R-M, and R-H: the concentrations of red honeysuckle were 400, 800, and 1,000 μg/mL, respectively; *n* = 3; #*p* < 0.05, ##*p* < 0.01, compared to the control group; **p < 0.01 compared to the model group.

Using the CCK-8 method (**Figures 4A, B**), the green and red honeysuckle aqueous extracts showed no cytotoxicity to RAW264.7 cells. All the cell viabilities of different groups were more than 100% when the drug concentrations were 12.5–1,000 μg/mL, which indicated that the two aqueous extracts were safe to a certain extent.

Moreover, the anti-inflammatory effects on LPS-induced RAW264.7 cells are shown in **Figures 4C–I**. NO secretion results (**Figure 4C**) showed that compared with the control group, when LPS activated inflammatory response, the concentration of NO in the model group increased to 19.29 μM significantly. However, the concentrations of NO in the administration groups decreased significantly (*p* < 0.01), which indicated that the two honeysuckle aqueous extracts could inhibit the secretion of NO and had certain anti-inflammatory effects, with a certain concentration dependence. Additionally, the inhibitory effect of red honeysuckle was stronger than that of the traditional green honeysuckle.

The concentrations of major inflammatory factors IL-6, TNF-α, and IL-1β in the supernatant are shown in **Figures 4D–F**. After LPS induction, they were significantly higher in the model group than in the control group, and inflammation occurred. Their concentrations decreased and showed a certain concentration-dependent relationship (*p* < 0.01). Moreover, the relative mRNA expressions of IL-6, TNF-α, and IL-1β were measured by rt-qPCR, and the results are shown in **Figures 4G–I**. Similarly, when LPS was induced, the expression of inflammatory factors increased in varying degrees. There was a significant increase in the model group when compared with the control. Their expressions in G-L, G-M, G-H, R-L, R-M, and R-H were decreased after administration (*p* < 0.01). Interestingly, these two extracts showed clear concentration-dependent relationships. The higher the drug concentration, the lower levels of mRNA expression.

These results suggested that two honeysuckle aqueous extracts could inhibit the expression of NO, IL-6, TNF-α, and IL-1β induced by LPS dose dependently, which was consistent with previous studies (Guo et al., 2021; Su et al., 2021). It also showed that they had good anti-inflammatory activity, and red honeysuckle showed better inhibitory effect.

### 3.4 Bio-active component analysis

**Figure 5:**
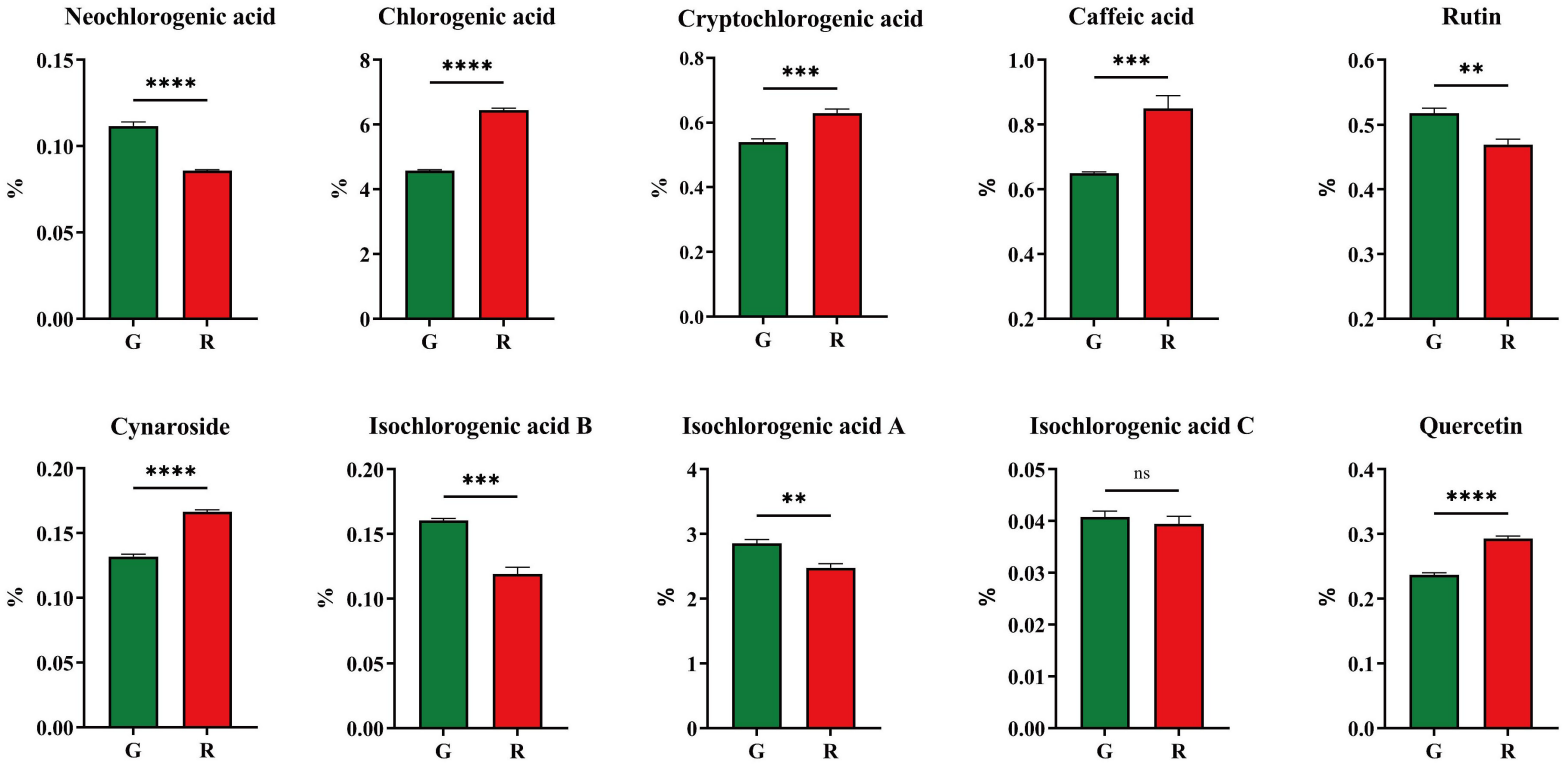
Ten main compounds of honeysuckles. G, green honeysuckle; R, red honeysuckle. ns, no significance, **p ≤ 0.01, ***p ≤ 0.001, ****p ≤ 0.0001.

The results of physio−chemical indicators showed that there might be a significant difference in chemical composition. In this study, 10 bio-active components were analyzed and their content is shown in **Figure 5**. Compared with green honeysuckle, red honeysuckle had a lower content of neochlorogenic acid, rutin, and isochlorogenic acids A, B, and C, and a higher content of chlorogenic acid, cryptochlorogenic acid, caffeic acid, cynaroside, and quercetin. There were significant differences in these components except for isochlorogenic acid C. For chlorogenic acid, its content (6.45% ± 0.06%) in red honeysuckle was higher than that (4.57% ± 0.03%) in green honeysuckle. Similarly, the average content of cynaroside (0.16%) in red honeysuckle was higher than that (0.13%) in green honeysuckle. It was indicated that red honeysuckle had higher medicinal value. In particular, the quercetin content of red honeysuckle was significantly higher (*p* ≤ 0.0001) than that of green honeysuckle.

### 3.5 Volatile metabolite analysis

#### 3.5.1 Metabolite profiling analysis

The separate (**Supplementary Figure S1A**) and overlaid (**Supplementary Figure S1B**) analysis of the total ion chromatogram (TIC) showed that the instrument had a good stability, which improved repeatability and reliability.

**Figure 6:**
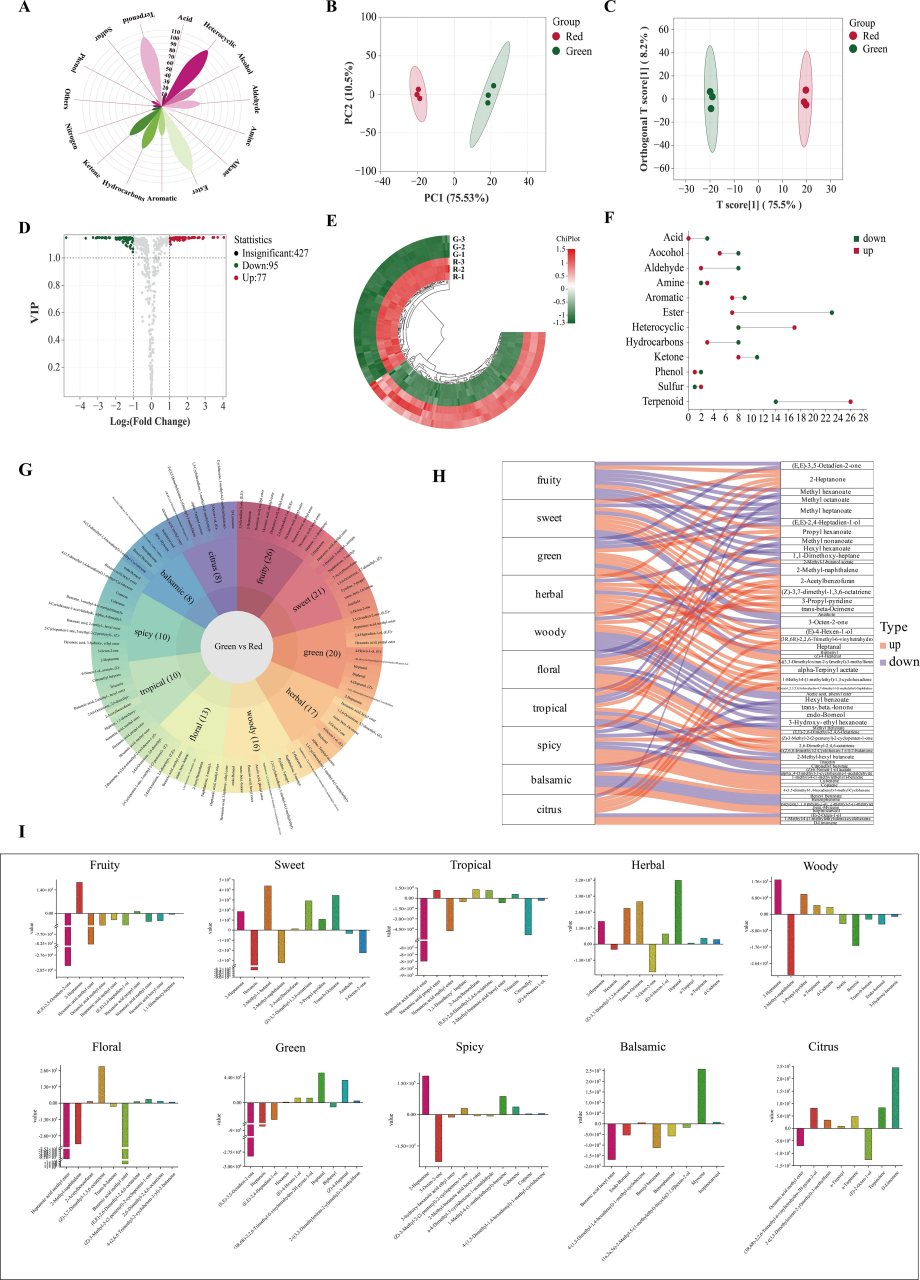
Volatile metabolite analysis. **(A)** Classification of all metabolites. **(B)** PCA score plot. **(C)** OPLS−DA score plot. **(D)** Volcano plot. **(E)** Heatmap of differential metabolites; **(F)** Up− and downregulation of differential metabolites. **(G)** Flavor sunburst. **(H)** Flavor sankey. **(I)** Metabolite content variation among flavors.

A total of 605 volatile metabolites were identified (**Figure 6A**), namely, 103 terpenoids, 102 heterocyclic compounds, 100 esters, 62 hydrocarbons, 56 ketones, 48 aldehydes, 48 alcohols, 36 aromatics, 13 amines, 11 acids, 8 phenols, 6 nitrogen compounds, 4 halogenated hydrocarbons, 4 sulfur compounds, and 4 others.

#### 3.5.2 PCA and OPLS−DA

Principal component analysis (PCA) could preserve as much of the original information as possible with fewer comprehensive variables (Clark et al., 2019). Based on these metabolites, a non-supervised PCA model was used for comprehensive analysis. The total contribution rate of 86.03% indicated the validity and stability of this model. As shown in **Figure 6B**, green and red honeysuckles could be distinguished well, which indicated that their volatile metabolites were significantly different.

However, PCA cannot ignore within-group errors (Liu et al., 2022). Therefore, supervised orthogonal partial least squares discrimination analysis (OPLS−DA) was used to further analyze the difference of this variables. It is known that *Q* ^2^ is an important parameter in determining the predictive capability of the model. The model is excellent and stable when *Q* ^2^ is >0.9. The validity of the OPLS−DA model was assessed due to high *Q* ^2^ (0.991), *R* ^2^ *X* (0.756), and *R* ^2^ *Y* (0.998) (**Supplementary Figures S2A, B**). In **Figure 6C**, it could be seen that green and red honeysuckles were clearly distinguished and this model was stable, reliable, and effective. The above result provided an excellent explanation for the metabolic variations.

#### 3.5.3 Differential metabolite screening

The above results revealed the different accumulation patterns of volatile metabolites. To further identify the differential volatile metabolites between green and red honeysuckles, 605 metabolites were analyzed according to the principle of variable important in projection (VIP) > 1 and |log_2_(fold change)| ≥ 1 (**Figure 6D**). A total of 178 differential metabolites were screened out (**Supplementary Table S2**), of which 81 metabolites such as Δ−limonene, copaene, and (*Z*)−4−heptenal were upregulated, but 97 metabolites such as benzyl benzoate, methyl dodecanoate, and 3−nonen−5−one were downregulated (**Figure 6E**).

These differential metabolites were classified into 12 categories (**Figure 6F**). Most of the upregulated metabolites were terpenoids (32.10%) and heterocyclic compounds (20.99%), and most of the downregulated metabolites were esters (23.71%) and terpenoids (14.43%). This comparative analysis revealed that heterocyclic compounds were significantly more abundant and esters were significantly less abundant in red honeysuckle than those in green honeysuckle. For terpenoids, their upregulation and downregulation in red honeysuckle might lead to a significant flavor difference.

**Figure 7:**
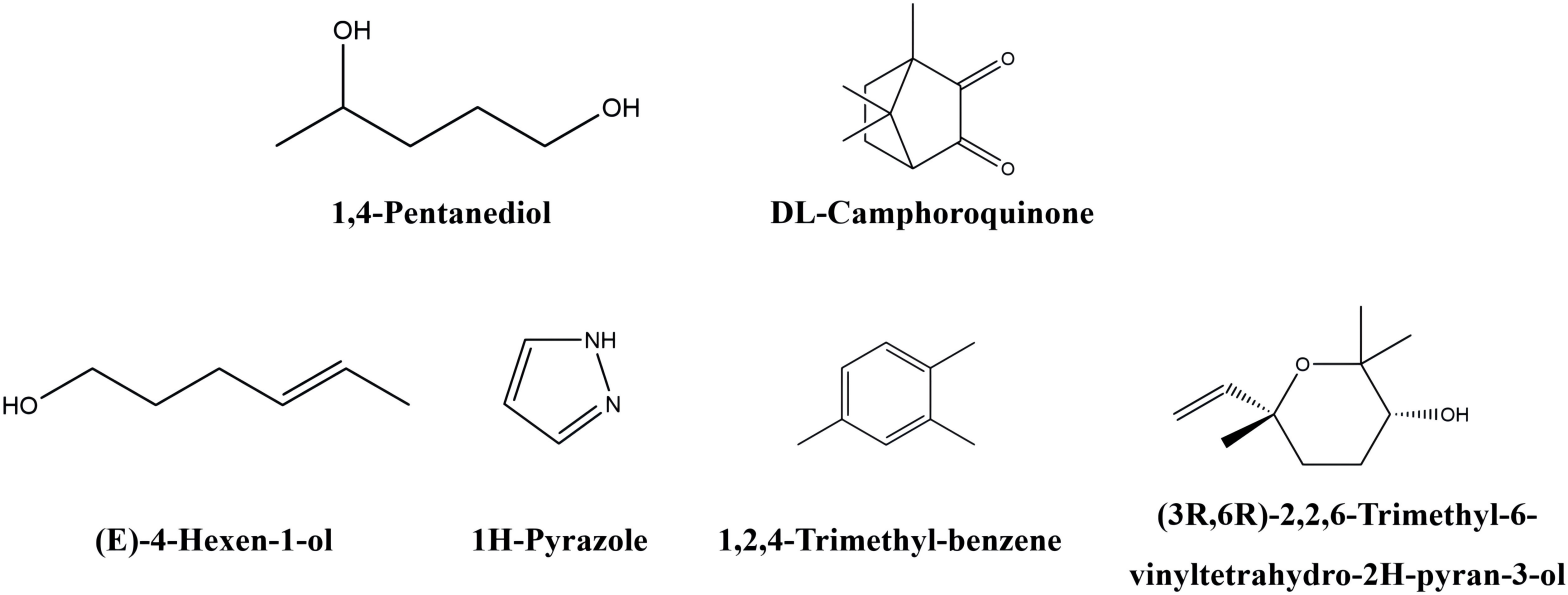
The chemical structure of unique volatile metabolites in green and red honeysuckles.

As shown in **Table 2** and **Figure 7**, some differential metabolites were found only in green or red honeysuckles. For instance, 1,4−pentanediol and DL−camphoroquinone were only detected in green honeysuckle, and (*E*)−4−hexen−1−ol,pyrazole, 1,2,4−trimethyl-benzene, and (3R,6R)−2,2,6−trimethyl−6−vinyltetrahydro−2H−pyran−3−ol were only detected in red honeysuckle.

**Table 2:** Unique volatile metabolites of green and red honeysuckle.

Group	Compounds	Class	Formula	CAS
Green	1,4-Pentanediol	Alcohol	C5H12O2	626-95-9
	dl-Camphoroquinone	Terpenoids	C10H14O2	10373-78-1
Red	(*E*)-4-Hexen-1-ol	Alcohol	C6H12O	928-92-7
	Pyrazole	Heterocyclic compound	C3H4N2	288-13-1
	1,2,4-Trimethyl-benzene	Aromatics	C9H12	95-63-6
	(3*R*,6*R*)-2,2,6-Trimethyl-6-vinyltetrahydro-2H-pyran-3-ol	Heterocyclic compound	C10H18O2	14009-71-3

#### 3.5.4 Sensory flavor analysis

Aroma is closely related to the taste and nutritional quality of plants and foods and is an important factor affecting its characteristics (Banožić et al., 2020). According to sensory analysis, differential metabolites can be characterized based on their flavor characteristics. In this study, the sensory flavor characteristics of 178 differential metabolites were annotated (**Figure 6G**). The top 10 sensory flavor characteristics of differential metabolites were fruity, sweet, green, herbal, woody, floral, tropical, spicy, balsamic, and citrus. Fruity, sweet, and green were annotated by 26, 21, and 20 differential metabolites, respectively.

The association was analyzed between the top 10 differential metabolites (ranked by VIP value) and the corresponding flavor characteristics (**Figure 6H**). For downregulated metabolites, (*E*,*E*)−3,5−octadien−2−one decreased fruity and green; methyl heptanoate decreased fruity, sweet, green, and floral; and 2−methyl−naphthalene decreased fruity, sweet, green, and floral. For upregulated metabolites, 2−heptanone increased fruity, sweet, herbal, woody, and spicy; propyl hexanoate increased fruity, green, and tropical; and (*Z*)−3,7−dimethyl−1,3,6−octatriene increased sweet, herbal, and floral.

Each flavor characteristic was associated with both up− and downregulated metabolites; thus, it was impossible to assess the final difference. Therefore, it was necessary to study the specific content difference of each metabolite corresponding to the same flavor. In **Figure 6I**, red honeysuckle had stronger herbal, spicy, and citrus flavors, and lighter fruity, sweet, green, woody, floral, tropical, and balsamic flavors. These results fully explained the reason why the herbal flavor of red honeysuckle was stronger than that of green honeysuckle.

### 3.6 Non-volatile metabolite analysis

#### 3.6.1 Metabolite profiling analysis

The TIC of green and red honeysuckles was performed in both positive and negative ion modes. The results showed the characteristic metabolites of both red and green honeysuckles and are found in **Supplementary Figures S3A, B**. It could be seen that the signal stability was good according to the overlap TIC of QC samples (**Supplementary Figures S3C, D**), which provided an important guarantee for repeatability and reliability.

**Figure 8:**
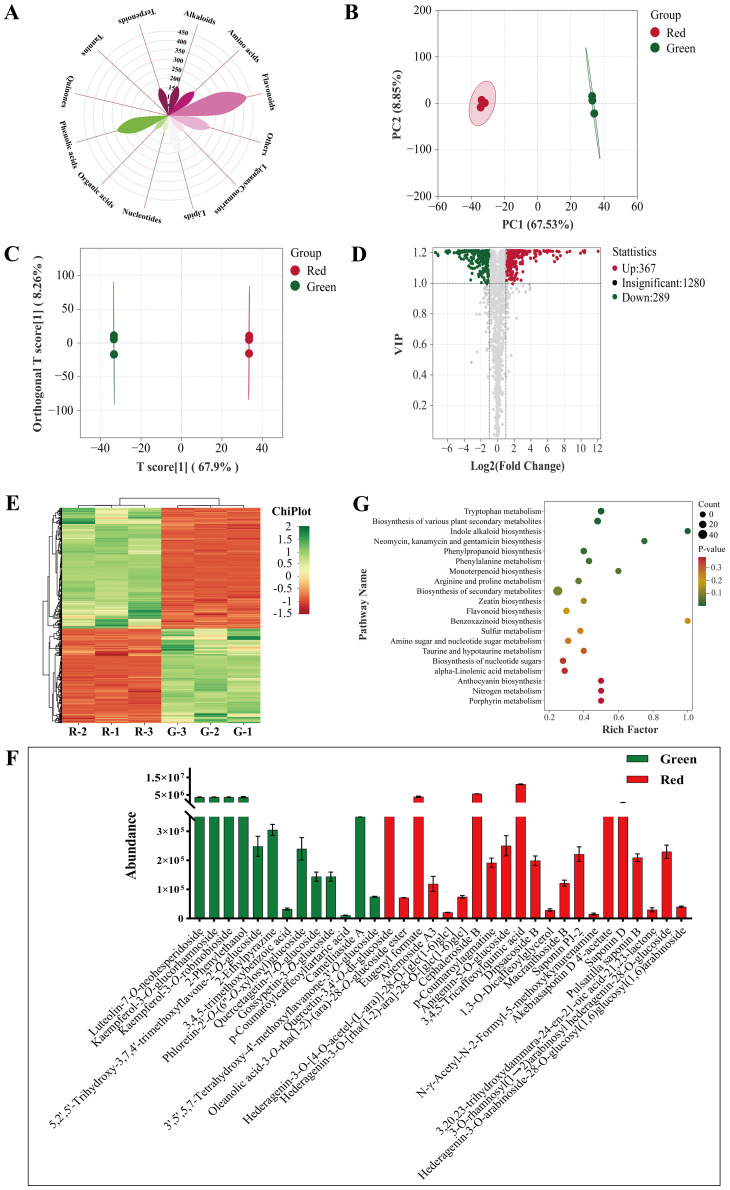
Non-volatile metabolite analysis. **(A)** Classification of all metabolites. **(B)** PCA score plot. **(C)** OPLS−DA score plot. **(D)** Volcano plot. **(E)** Heatmap of differential metabolites. **(F)** Specific metabolites. **(G)** KEGG enrichment analysis.

There were 1,971 non−volatile metabolites identified (**Figure 8A**), namely, 426 flavonoids, 284 phenolic acids, 205 lipids, 186 amino acid and derivatives, 161 alkanoids, 153 terpenoids, 120 lignans and coumarins, 100 organic acids, 89 nucleotides and derivatives, 12 quinones, 6 tannins, and 229 others.

#### 3.6.2 PCA and OPLS−DA

The PCA score (**Figure 8B**) showed good separation and indicated a significant difference. The permutation plot of OPLS−DA showed that this model had effective predictive ability and did not experience overfitting (**Supplementary Figures S4A, B**). The OPLS−DA score plots (**Figure 8C**) also showed significant differences similar to PCA.

#### 3.6.3 Differential metabolite screening

VIP > 1 and |log_2_(fold change)| ≥ 1 were used as reference to screen different metabolites (**Figure 8D**). A total of 691 differential metabolites were screened out (**Figure 8E**). Flavonoids and phenolic acids accounted for 25.04% and 15.77% in honeysuckle, respectively. There were 388 upregulated metabolites such as peonidin−3−*O*−glucoside, vanillic acid, and tryptamine, and 303 downregulated metabolites such as secologanin, methyl *p*−coumarate, and geniposidic acid.

Notably, the content of procyanidin B5, a proanthocyanidin metabolite, in green honeysuckle was significantly higher (*p* ≤ 0.05) than that in red honeysuckle. In contrast, the content of peonidin−3−*O*−glucoside, an anthocyanin metabolite, in red honeysuckle was significantly higher (*p* ≤ 0.05) than that in green honeysuckle. Plants produce proanthocyanidins as polyphenols in their flowers, fruit cores, and leaves. Anthocyanins are water−soluble natural pigments in plants. As a result of their excellent anti-oxidation properties and health benefits such as preventing cancer, protecting vision, slowing aging, and beautifying the skin, proanthocyanidins and anthocyanins are becoming more popular (Zhao et al., 2023). Anthocyanins are colored such as purple centaurin, orange geranium, and blue−purple delphinium, but proanthocyanidins are colorless (Khoo et al., 2017). Peonidin−3−*O*−glucoside, a red anthocyanin metabolite, is methylated centaurea. In this study, it was considered to be a key metabolite to show the significant difference between green and red honeysuckles due to its high accumulation pattern. In addition, this study has demonstrated its ability to induce anabolic impacts on bone by increasing osteoblast proliferation and differentiation and altering the osteoblast epigenome (Ren et al., 2021). Therefore, it is suggested that the red honeysuckle can be further developed into a series of osteoporosis medicines and health products.

Moreover, the content of some differential metabolites in red honeysuckle was higher than that in green honeysuckle, and their pharmacological activities significantly enhance the development value of red honeysuckle. For instance, verbascoside has been shown to alleviate pneumococcal pneumonia and inhibit PLY-mediated cytotoxicity (Zhao et al., 2016). It could also effectively inhibit the activity of SARS-CoV-2 main protease and protect against COVID-19 (Xiao et al., 2022). Similarly, gallocatechin has significant anti-inflammatory activity in a pleurisy model in mice (Siebert et al., 2021). Protocatechuic acid methyl ester has been found to effectively attenuate the F-induced changes in oxidative stress, inflammation, and apoptosis markers (Ameeramja and Perumal, 2017; Ameeramja et al., 2018). For other different metabolites, the content of red honeysuckle can also effectively indicate that the red honeysuckle has stronger antibacterial, anti-inflammatory, and antioxidant activities.

**Figure 9:**
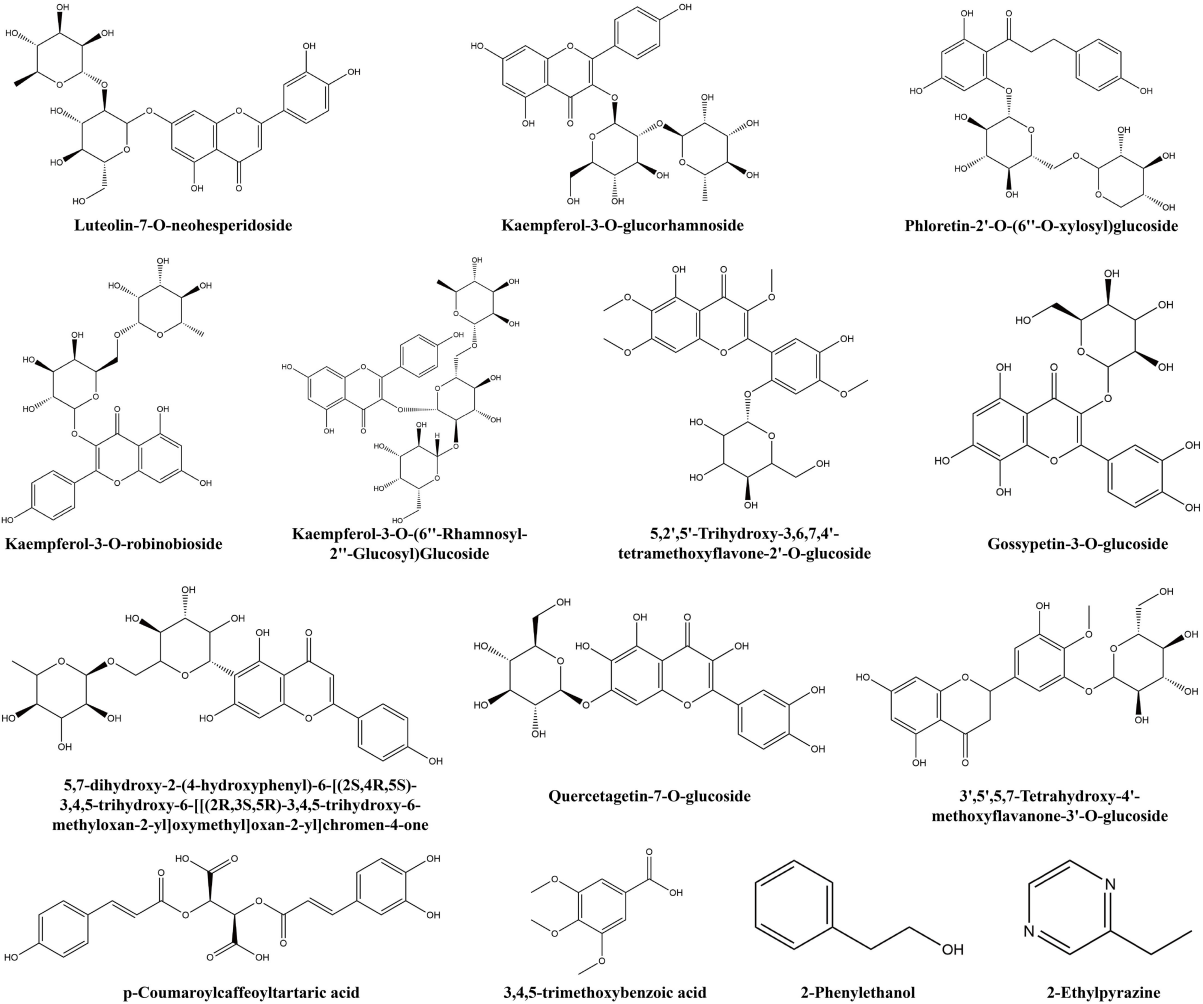
The chemical structure of unique non-volatile metabolites of green honeysuckle.

**Figure 10:**
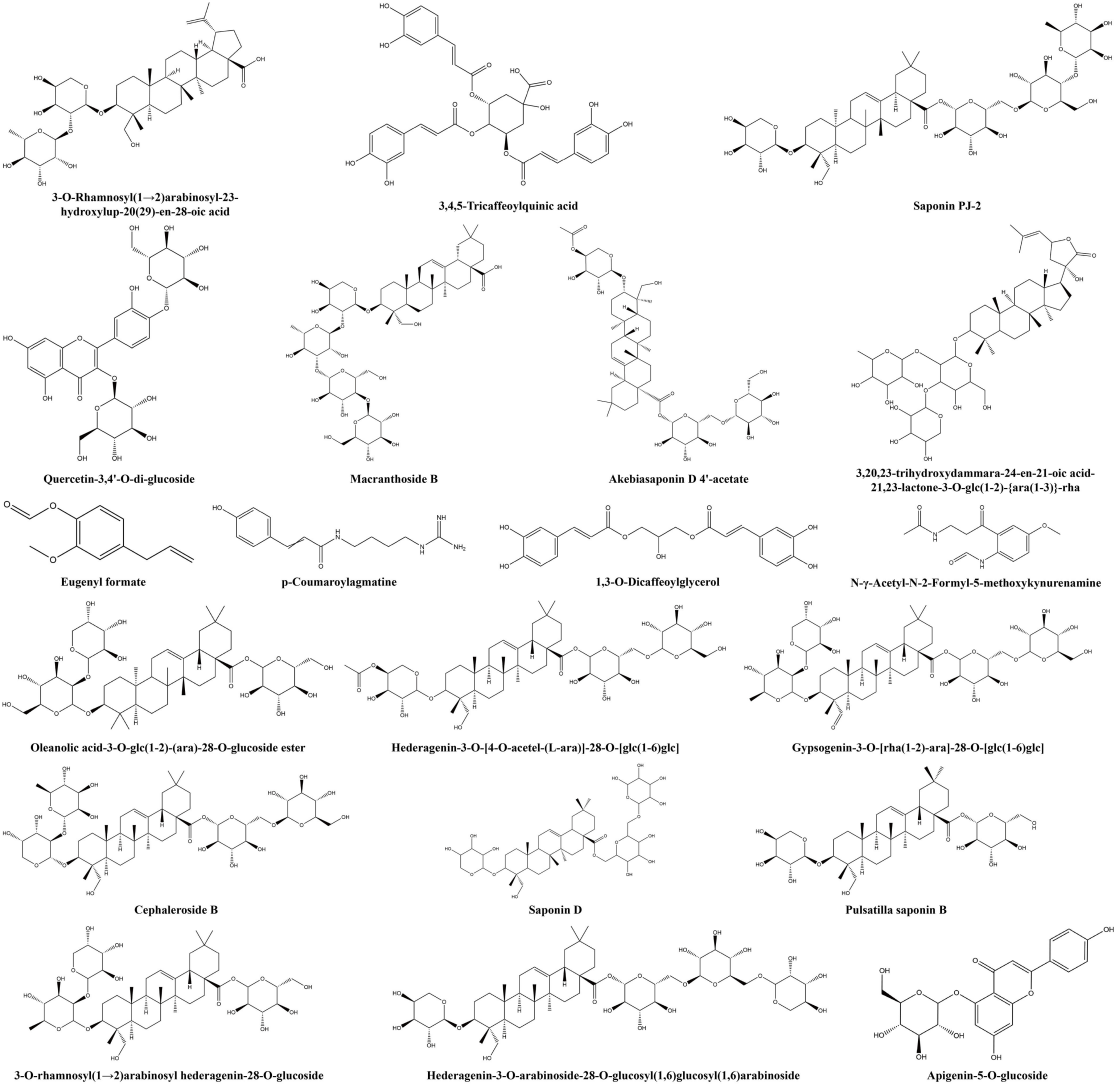
The chemical structure of unique non-volatile metabolites of red honeysuckle.

Additionally, some non−volatile metabolites were found only in green or red honeysuckles (**Table 3**). For example, there were 14 differential metabolites (such as lonicerin, biorobin, and kaempferol−3−*O*−glucorhamnoside) found only in green honeysuckle and 21 differential metabolites (such as quercetin−3,4’−*O*−di−glucoside, cephaleroside B, and apigenin−5−*O*−glucoside) found only in red honeysuckle. Their relative contents are shown in **Figure 8F**. Their chemical structures are shown in **Figures 9** and **10**, respectively. It is because of these unique metabolites that the difference in efficacy and function of green and red honeysuckles is recognized.

**Table 3:** Unique non−volatile metabolites of green or red honeysuckle.

Group	Compounds	Class	Formula	CAS
Green	Luteolin-7-*O*-neohesperidoside	Flavonoids	C27H30O15	25694-72-8
	Kaempferol-3-*O*-glucorhamnoside	Flavonoids	C27H30O15	40437-72-7
	Kaempferol-3-*O*-robinobioside	Flavonoids	C27H30O15	17297-56-2
	2-Phenylethanol	Phenolic acids	C8H10O	60-12-8
	5,2',5'-Trihydroxy-3,6,7,4'-tetramethoxyflavone-2'-*O*-glucoside	Flavonoids	C25H28O14	–
	2-Ethylpyrazine	Alkaloids	C6H8N2	13925-00-3
	3,4,5-Trimethoxybenzoic acid	Phenolic acids	C10H12O5	118-41-2
	5,7-Dihydroxy-2-(4-hydroxyphenyl)-6-[(2S,4R,5S)-3,4,5-trihydroxy-6-[[(2R,3S,5R)-3,4,5-trihydroxy-6-methyloxan-2-yl]oxymethyl]oxan-2-yl]chromen-4-one	Flavonoids	C27H30O14	–
	Phloretin-2'-*O*-(6''-*O*-xylosyl)glucoside	Flavonoids	C26H32O14	–
	Quercetagetin-7-*O*-glucoside	Flavonoids	C21H20O13	548-75-4
	Gossypetin-3-*O*-glucoside	Flavonoids	C21H20O13	777080-67-8
	*p*-Coumaroylcaffeoyltartaric acid	Phenolic acids	C22H18O11	–
	Kaempferol-3-*O*-(6''-rhamnosyl-2''-glucosyl)glucoside	Flavonoids	C33H40O20	135095-52-2
	3',5',5,7-Tetrahydroxy-4'-methoxyflavanone-3'-*O*-glucoside	Flavonoids	C22H24O12	–
Red	Quercetin-3,4'-*O*-di-glucoside	Flavonoids	C27H30O17	29125-80-2
	Oleanolic acid-3-*O*-glc(1-2)-(ara)-28-*O*-glucoside ester	Terpenoids	C47H76O17	–
	Eugenyl formate	Others	C11H12O3	10031-96-6
	3-*O*-Rhamnosyl(1→2)arabinosyl-23-hydroxylup-20(29)-en-28-oic acid	Terpenoids	C41H66O12	129724-84-1
	Hederagenin-3-*O*-[4-*O*-acetel-(*L*-ara)]-28-*O*-[glc(1-6)glc]	Terpenoids	C49H78O19	–
	Gypsogenin-3-*O*-[rha(1-2)-ara]-28-*O*-[glc(1-6)glc]	Terpenoids	C53H84O22	–
	Cephaleroside B	Terpenoids	C53H86O22	–
	*p*-Coumaroylagmatine	Alkaloids	C14H20N4O2	7295-86-5
	Apigenin-5-*O*-glucoside	Flavonoids	C21H20O10	28757-27-9
	3,4,5-Tricaffeoylquinic acid	Phenolic acids	C34H30O15	86632-03-3
	Dipsacoside B	Terpenoids	C53H86O22	33289-85-9
	1,3-*O*-Dicaffeoylglycerol	Phenolic acids	C21H20O9	–
	Macranthoside B	Terpenoids	C53H86O22	146100-02-9
	Saponin PJ-2	Terpenoids	C53H86O22	–
	N-*γ*-Acetyl-*N*-2-formyl-5-methoxykynurenamine	Amino acids and derivatives	C13H16N2O4	52450-38-1
	Akebia saponin D 4'-acetate	Terpenoids	C49H78O19	126778-93-6
	Akebia saponin D	Terpenoids	C47H76O18	39524-08-8
	3-*O*-α-*L*-Arabinopyranosylhederagenin-28-β-*D*-glucopyranoside	Terpenoids	C41H66O13	39524-13-5
	3,20,23-Trihydroxydammara-24-en-21-oic acid-21,23-lactone-3-O-glc(1-2)-{ara(1-3)}-rha	Terpenoids	C47H76O17	–
	3-*O*-rhamnosyl(1→2)arabinosyl hederagenin-28-*O*-glucoside	Terpenoids	C47H76O17	–
	Hederagenin-3-*O*-arabinoside-28-*O*-glucosyl(1,6)glucosyl(1,6)arabinoside	Terpenoids	C52H84O22	–

#### 3.6.4 KEGG classification and enrichment analysis

Plants and foods contain metabolites that coordinate with one another and carry out biological functions together. Analyzing and annotating their metabolic pathways could further explain their corresponding functions. In this study, 691 differential non-volatile metabolites were mapped to the KEGG database and carried out pathway enrichment analysis. In **Supplementary Figure S4C**, these metabolites were annotated to 65 metabolic pathways that were mainly involved in three primary functions: metabolism, environmental information processing, and genetic information processing. These pathways promoted biosynthesis of secondary metabolites, biosynthesis of secondary metabolites, and tryptophan metabolism (**Supplementary Figure S4D**). Cluster analysis was performed for all differential metabolites in enriched KEGG metabolic pathways. In **Figure 8G**, compared with green honeysuckle, red honeysuckle mainly enhanced tryptophan metabolism, phenylpropanoid biosynthesis, and various plant secondary metabolites’ biosynthesis due to the high accumulation pattern of differential metabolites such as tryptamine, caffeic acid, and 2−hydroxycinnamic acid.

### 3.7 Correlation analysis of differential metabolites

**Figure 11:**
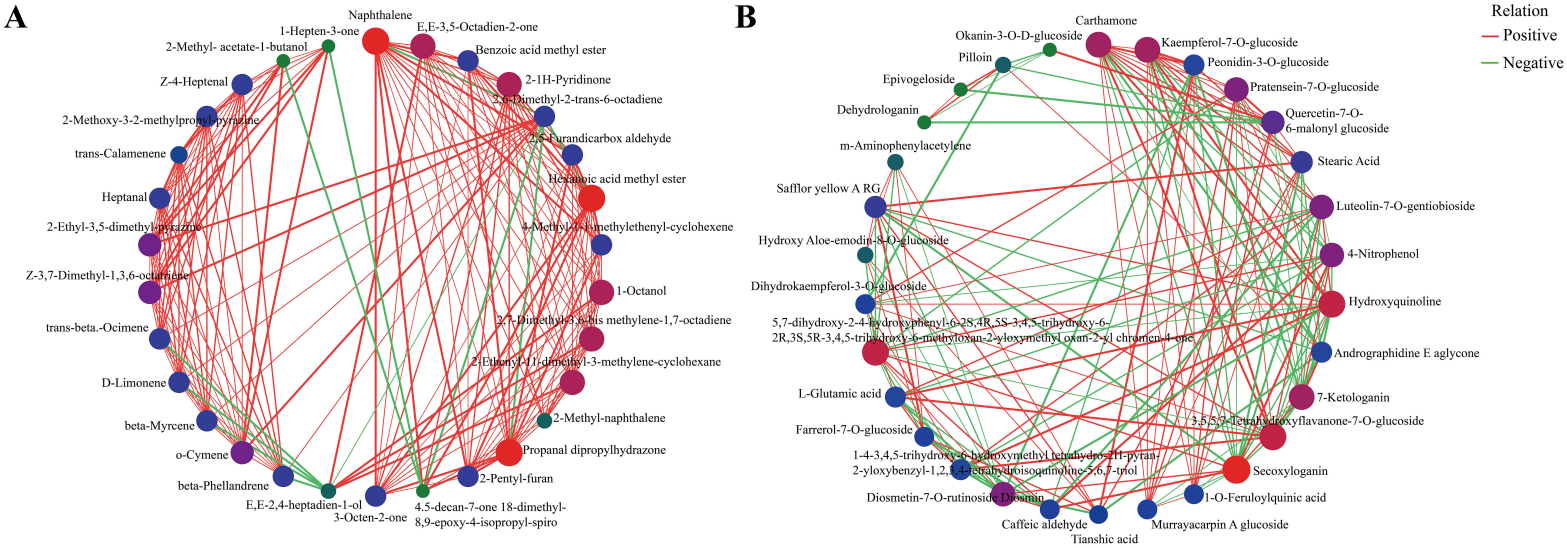
Correlation network plots for significant differential metabolites (top 30) of green and red honeysuckles. **(A)** Volatile metabolites. **(B)** Non−volatile metabolites.

Significant differential metabolic relationships were investigated using Spearman’s paired rank correlation analysis, and the molecular networks are shown in **Figure 11A** (volatile metabolites) and **Figure 11B** (non−volatile metabolites). Red, blue, and green dots indicated strong, medium, and weak correlations, respectively. The larger the dot, the more related the metabolites are. Positive and negative correlations may represent synthesis and conversion, respectively (Markkinen et al., 2022).

As shown in **Figure 11A**, most of the correlations were positive. For example, (*E*,*E*)−2,4−heptadien−1−ol was positively correlated with 1−octanol, 2,7−dimethyl−3,6−bis(methylene)−1,7−octadiene, and 2−ethenyl−1,1−dimethyl−3−methylene−cyclohexane, and negatively correlated with β−hellandrene, β−myrcene, Δ−limonene, and trans−β−ocimene. In **Figure 11B**, carthamone, kaempferol−7−*O*−glucoside, and peonidin−3−*O*−glucoside were the top three metabolites that showed the most correlation with other metabolites. Kaempferol−7−*O*−glucoside, an important flavonoid compound of honeysuckle, has anti−HSV activity and can be considered as a new potential drug candidate for the treatment of HIV infection (Tang et al., 2024). Peonidin−3−*O*−glucoside can not only increase osteoblast differentiation but also serve as a virtual predictor of the anti-inflammatory activity of TNF−α signaling (Ren et al., 2021). These results again showed the potential application value of red honeysuckle.

## 4 Discussion

The determination of main biological activities was the main entry point for most scholars to study the targets and mechanisms of honeysuckle in the treatment of diseases (Yang et al., 2014; Lv et al., 2021; Yeh et al., 2022). As more and more plants are found to have antioxidant properties, there is increasing interest in measuring total antioxidant activity rather than simply the contents of different antioxidants (Ramón-Sierra et al., 2022). In this study, red honeysuckle had a higher activity based on three essays, which indicated its potential in cosmetics, pharmaceuticals, and functional foods. Moreover, red honeysuckle also had a higher quercetin content, which further demonstrated its development value.

Similarly, *in vitro* experiments, including cell activity and gene expression, proved that red honeysuckle had higher antibacterial and anti-inflammatory effects. Because its aqueous extracts could inhibit the growth of *S. aureus*, *E. coli*, and *B. subtilis*, producers could use the extract of honeysuckle to prepare AgNP-coated silks for colorful and long-term multifunctional textiles (Zhou and Tang, 2018). In terms of cytotoxicity, our study found that neither of the two water extracts was toxic to RAW264.7 at the same concentration. The traditional green honeysuckle has been recognized as safe, reliable, and edible (Kucharska et al., 2017; Ma et al., 2022). The safety of red honeysuckle, which can be used as a food raw material, is also shown. Furthermore, its aqueous extract could inhibit the expression of IL-6, IL-1β, and TNF-α at both cellular and gene levels. It was found that this inhibition may be due to the *in situ* enzymatic hydrolysis of RAW264.7 cells (Cao et al., 2018). On the whole, red honeysuckle could reduce inflammation by controlling inflammatory factors.

In addition, the bio-active components chlorogenic acid has a series of biological activities at the gastrointestinal level, in addition to decreasing inflammatory cytokines by inhibiting the NF-B pathway (Luo et al., 2021; La Rosa et al., 2023). Cynaroside could reduce body weight, total cholesterol, triglyceride, and LDL-C levels; increase blood HDL-C levels; and reduce liver steatosis (Sun et al., 2021). Caffeic acid could control MAPK and NF-κB activation and significantly reduce serum TNF-α and IL-6 levels when administered during LPS stimulation (Choi et al., 2017). Furthermore, studies have found that quercetin and its derivative quercetin caprylate are proteasome activators that have anti-aging and antioxidant properties (Chondrogianni et al., 2010). Interestingly, compared with green honeysuckle, red honeysuckle had a higher content of these compounds, which were significantly higher than the relevant limit in Chinese Pharmacopoeia. These results provided data support for the application of red honeysuckle in medicines, functional foods, and cosmetics from the perspective of biological activity.

For metabolites, many terpenoids with herbal flavor such as copaene, β−ocimene, and δ−cadinene were identified, and their content was significantly higher in red honeysuckle than in green honeysuckle. Some upregulated metabolites such as 2−heptanone and heptanal and specific metabolites such as (*E*)−4−hexen−1−ol and 1,2,4−trimethyl−benzene also have a herbal flavor. These compounds were the material basis of the rich aroma of red honeysuckle, and were also the main reason why the public prefers it. Moreover, 67% of the specific non-volatile metabolites of red honeysuckle were triterpenoid saponins, which were regarded as potential antitumor agents. For example, dipasperoside B has shown inhibitory activity against NO production in an LPS-stimulated murine macrophage RAW264.7 cell line (Li et al., 2016). Macranthoside B could inhibit cell growth especially in human acute promyelocytic leukemia HL-60 cells (Guan et al., 2011). Akebia saponin D could significantly decrease NO production and iNOS expression, and has anti-nociceptive and anti-inflammatory effects (Gong et al., 2019). These findings provided sufficient theoretical basis for the development of red honeysuckle in health products.

As for the metabolic pathway, some differential metabolites were mainly enriched in tryptophan metabolism pathway by KEGG analysis. Six upregulated differential metabolites related to this pathways were valine−tryptophan, *N*−(1−deoxy−1−fructosyl)−tryptophan, *L*−tryptophan, 5−hydroxy−*L*−tryptophan, glycyl−tryptophan, and *N*−acetyl−*L*−tryptophan. Tryptophan (Trp) is an essential amino acid and plays an important role in physiology and pathophysiology (Seo and Kwon, 2023). The results revealed the extensive development potential of red honeysuckle from another dimension. On the whole, the data obtained in this study could prove that red honeysuckle was better than green honeysuckle, and it was worthy of comprehensive and in-depth development and utilization.

## 5 Conclusion

In this study, compared with traditional green honeysuckle, the advantages and resource development potential of red honeysuckle were explored. This study provided novel evidence of the quality characterization and material basis of the unique flavor of red honeysuckle. Furthermore, combined with its safe properties, bright color, and effective biological activities, it can be used as a raw material that can be processed into medicines, foods, beverages, biscuits, pigment additives, and even functional foods in the future. In conclusion, our study contributed to reveal the potential applications of red honeysuckle and could help advance such applications.

## Data Availability

The original contributions presented in the study are included in the article/**Supplementary Material**. Further inquiries can be directed to the corresponding author.

## References

[B1] Ameeramja J. Kanagaraj V. V. Perumal E. (2018). Protocatechuic acid methyl ester modulates fluoride induced pulmonary toxicity in rats. Food. Chem. Toxicol. 118, 235–244. doi: 10.1016/j.fct.2018.05.031 29758312

[B2] Ameeramja J. Perumal E. (2017). Protocatechuic acid methyl ester ameliorates fluoride toxicity in A549 cells. Food. Chem. Toxicol. 109, 941–950. doi: 10.1016/j.fct.2016.12.024 28012895

[B3] Banožić M. Jokić S. Ačkar Đ. Blažić M. Šubarić D. (2020). Carbohydrates-key players in tobacco aroma formation and quality determination. Molecules 25, 1734. doi: 10.3390/molecules25071734 32283792 PMC7181196

[B4] Belyaeva O. V. Sergeeva I. Y. Belyaeva E. E. Chernobrovkina E. V. (2021). Study of antioxidant activity of juices and beverages from blue honeysuckle and black chokeberry. Iop Conf. Series. Earth Environ. Sci. 640, 52008. doi: 10.1088/1755-1315/640/5/052008

[B5] Cao Y. Zhou X. Yin Z. Yu X. Yang Q. Guo Q.. (2018). The anti-inflammatory effect of BML-111 on COPD may be mediated by regulating NLRP3 inflammasome activation and ROS production. Prostaglandins Other Lipid Mediat. 138, 23–30. doi: 10.1016/j.prostaglandins.2018.08.001 30102962

[B6] Carrillo-Galván G. Bye R. Eguiarte L. E. Cristians S. Pérez-López P. Vergara-Silva F.. (2020). Domestication of aromatic medicinal plants in Mexico: Agastache (Lamiaceae)-an ethnobotanical, morpho-physiological, and phytochemical analysis. J. Ethnobiol. Ethnomed. 16, 22. doi: 10.1186/s13002-020-00368-2 32357896 PMC7193375

[B7] Choi K. C. Son Y. O. Hwang J. M. Kim B. T. Chae M. Lee J. (2017). Antioxidant, anti-inflammatory and anti-septic potential of phenolic acids and flavonoid fractions isolated from *Lolium* multiflorum. Pharm. Biol. 55, 611–619. doi: 10.1080/13880209.2016.1266673 27937124 PMC6130696

[B8] Chondrogianni N. Kapeta S. Chinou I. Vassilatou K. Papassideri I. Gonos E. S. (2010). Anti-ageing and rejuvenating effects of quercetin. Exp. Gerontol. 45, 763–771. doi: 10.1016/j.exger.2010.07.001 20619334

[B9] Clark C. C. T. Barnes C. M. Duncan M. J. Summers H. D. Stratton G. (2019). Physical activity, motor competence and movement and gait quality: A principal component analysis. Hum. Mov. Sci. 68, 102523. doi: 10.1016/j.humov.2019.102523 31683083

[B10] Eltaib L. Alzain A. A. (2023). Targeting the omicron variant of SARS-CoV-2 with phytochemicals from Saudi medicinal plants: molecular docking combined with molecular dynamics investigations. J. Biomol. Struct. Dyn 41, 9732–9744. doi: 10.1080/07391102.2022.2146203 36369836

[B11] Fang L. Long N. Li Y. Liao X. Shi L. Zhao H.. (2022). Transfer behavior of pesticides from honeysuckle into tea infusions: Establishment of an empirical model for transfer rate prediction. Ecotoxicol. Environ. Saf. 234, 113377. doi: 10.1016/j.ecoenv.2022.113377 35272189

[B12] Feng G. Li D. Liu J. Sun S. Zhang P. Liu W.. (2022). The herbal combination of *Radix astragali*, *Radix angelicae sinensis*, and *Caulis lonicerae* regulates the functions of type 2 innate lymphocytes and macrophages contributing to the resolution of collagen-induced arthritis. Front. Pharmacol. 13. doi: 10.3389/fphar.2022.964559 PMC9343953 35928276

[B13] Gong L. L. Yang S. Liu H. Zhang W. Ren L. L. Han F.F.. (2019). Anti-nociceptive and anti-inflammatory potentials of Akebia saponin D. Eur. J. Pharmacol. 845, 85–90. doi: 10.1016/j.ejphar.2018.11.038 30508505

[B14] Guan F. Shan Y. Zhao X. Zhang D. Wang M. Peng F.. (2011). Apoptosis and membrane permeabilisation induced by macranthoside B on HL-60 cells. Nat. Prod. Res. 25, 332–340. doi: 10.1080/14786411003752086 21328130

[B15] Guo X. Yu X. Zheng B. Zhang L. Zhang F. Zhang Y.. (2021). Network pharmacology-based identification of potential targets of *Lonicerae japonicae* flos acting on anti-inflammatory effects. BioMed. Res. Int. 2021, 5507003. doi: 10.1155/2021/5507003 34595237 PMC8478540

[B16] Hu Y. Li T. Gou Q. Zhang X. (2017). Determination of five active substances in different organs of *Lonicera japonica* var. *chinensis* (wats.) Bak. and *Lonicera japonica* Thunb. by HPLC method. J. Anhui Agric. Sci. 45, , 126–127,147. doi: 10.3969/j.issn.0517-6611.2017.34.040

[B17] Khoo H. E. Azlan A. Tang S. T. Lim S. M. (2017). Anthocyanidins and anthocyanins: Colored pigments as food, pharmaceutical ingredients, and the potential health benefits. Food Nutr. Res. 61, 1361779. doi: 10.1080/16546628.2017.1361779 28970777 PMC5613902

[B18] Kirina I. B. Titova L. V. Popova E. I. Grigoreva L. V. Khoroshkova Y. V. (2021). Biochemical value of berries of promising edible honeysuckle varieties for the production of functional food products. Iop Conf. Series. Earth Environ. Sci. 845, 12097. doi: 10.1088/1755-1315/845/1/012097

[B19] Kucharska A. Z. Sokol-Letowska A. Oszmianski J. Piorecki N. Fecka I. (2017). Iridoids, phenolic compounds and antioxidant activity of edible honeysuckle berries (*Lonicera caerulea* var. *Kamtschatica* sevast.). Molecules 22, 405. doi: 10.3390/molecules22030405 28273885 PMC6155291

[B20] La Rosa G. Sozio C. Pipicelli L. Raia M. Palmiero A. Santillo M.. (2023). Antioxidant, anti-inflammatory and pro-differentiative effects of chlorogenic acid on M03-13 human oligodendrocyte-like cells. Int. J. Mol. Sci. 24, 16731. doi: 10.3390/ijms242316731 38069054 PMC10706857

[B21] Li F. Tanaka K. Watanabe S. Tezuka Y. (2016). Dipasperoside B, a new trisiridoid glucoside from dipsacus asper. Nat. Prod. Commun. 11, 891–894. doi: 10.1177/1934578X1601100706 30452155

[B22] Li G. Liu J. Zhang H. Jia L. Liu Y. Li J.. (2023). Volatile metabolome and floral transcriptome analyses reveal the volatile components of strongly fragrant progeny of *Malus* × *robusta* . Front. Plant Sci. 14. doi: 10.3389/fpls.2023.1065219 PMC9895795 36743501

[B23] Li J. Jia G. Wang J. Li J. Yang L. (2013). Weight and index ingredients components content comparison of different flowering phase in *Lonicera japonica.* var. *Chinensis* (wats.) Bak. J. Henan Agric. Univ. 47, 534–537. doi: 10.3969/j.issn.1000-2340.2013.05.005

[B24] Li J. Ye C. Lian X. Wang L. Niu Y. (2019). Study on breeding system and pollination biology of *Lonicera japonica* var. *Chinensis* . J. Henan Agric. Univ. 53, 581–590. doi: 10.16445/j.cnki.1000-2340.2019.04.013

[B25] Liu Z. Cheng Y. Chao Z. (2023). A comprehensive quality analysis of different colors of medicinal and edible honeysuckle. Foods 12, 3126. doi: 10.3390/foods12163126 37628125 PMC10453482

[B26] Liu Z. Fang Y. Wu C. Hai X. Xu B. Li Z.. (2022). The difference of volatile compounds in female and male buds of herpetospermum pedunculosum based on HS-SPME-GC-MS and multivariate statistical analysis. Molecules 27, 1288. doi: 10.3390/molecules27041288 35209076 PMC8879731

[B27] Luo J. He W. Li X. Ji X. Liu J. (2021). Anti-acne vulgaris effects of chlorogenic acid by anti-inflammatory activity and lipogenesis inhibition. Exp. Dermatol. 30, 865–871. doi: 10.1111/exd.14277 33433016

[B28] Lv Q. Xing Y. Liu J. Dong D. Liu Y. Qiao H.. (2021). Lonicerin targets EZH2 to alleviate ulcerative colitis by autophagy-mediated NLRP3 inflammasome inactivation. Acta Pharm. Sin. B. 11, 2880–2899. doi: 10.1016/j.apsb.2021.03.011 34589402 PMC8463273

[B29] Ma A. Zou F. Zhang R. Zhao X. (2022). The effects and underlying mechanisms of medicine and food homologous flowers on the prevention and treatment of related diseases. J. Food Biochem. 46, e14430. doi: 10.1111/jfbc.14430 36165435

[B30] Markkinen N. Pariyani R. Jokioja J. Kortesniemi M. Laaksonen O. Yang B.. (2022). NMR-based metabolomics approach on optimization of malolactic fermentation of sea buckthorn juice with lactiplantibacillus plantarum. Food Chem. 366, 130630. doi: 10.1016/j.foodchem.2021.130630 34333181

[B31] Mu W. Hu N. Zhang L. Jiang W. Yan T. Zhang T.. (2022). *Lonicerae japonicae* flos ameliorates radiotherapy-induced mesenteric artery endothelial dysfunction through GTPCH_1_/BH_4_/eNOS pathway. Phytomedicine 102, 154146. doi: 10.1016/j.phymed.2022.154146 35594639

[B32] Ramón-Sierra J. M. Villanueva M. A. Yam-Puc A. Rodríguez-Mendiola M. Arias-Castro C. Ortiz-Vázquez E.. (2022). Antimicrobial and antioxidant activity of proteins isolated from *Melipona beecheii* honey. Food Chemistry: X 13, 100177. doi: 10.1016/j.fochx.2021.100177 35498960 PMC9039927

[B33] Ren Z. Raut N. A. Lawal T. O. Patel S. R. Lee S. M. Mahady G. B. (2021). Peonidin-3-*O*-glucoside and cyanidin increase osteoblast differentiation and reducerankl-induced bone resorption in transgenic medaka. Phytother. Res. 35, 6255–6269. doi: 10.1002/ptr.7271 34704297 PMC8942084

[B34] Seo S. Kwon B. (2023). Immune regulation through tryptophan metabolism. Exp. Mol. Med. 55, 1371–1379. doi: 10.1038/s12276-023-01028-7 37394584 PMC10394086

[B35] Siebert D. A. Paganelli C. J. Queiroz G. S. Alberton M. D. (2021). Anti-inflammatory activity of the epicuticular wax and its isolated compounds catechin and gallocatechin from *Eugenia brasiliensis* Lam. (Myrtaceae) leaves. Nat. Prod. Res. 35, 4720–4723. doi: 10.1080/14786419.2019.1710707 31913074

[B36] Su X. Zhu Z. Zhang L. Wang Q. Xu M. Lu C.. (2021). Anti-inflammatory property and functional substances of *Lonicerae japonicae* caulis. J. Ethnopharmacol. 267, 113502. doi: 10.1016/j.jep.2020.113502 33189843

[B37] Sun J. Wang Z. Chen L. Sun G. (2021). Hypolipidemic effects and preliminary mechanism of chrysanthemum flavonoids, its main components luteolin and luteoloside in hyperlipidemia rats. Antioxidants 10, 1309. doi: 10.3390/antiox10081309 34439559 PMC8389196

[B38] Tan Z. Lu D. Li L. Yu Y. Xu L. Dong W.. (2022). Loning and expression analysis of the anthocyanin synthase encoding gene RLJANS1 and its relationship with anthocyanin accumulation in honeysuckle. Jiangsu Agric. Sci. 50, 69–75. doi: 10.15889/j.issn.1002-1302.2022.21.010

[B39] Tang R. Lin L. Liu Y. Li H. (2024). Bibliometric and visual analysis of global publications on kaempferol. Front. Nutr. 11, 1442574. doi: 10.3389/fnut.2024.1442574 39221164 PMC11362042

[B40] Wang H. Hua J. Yu Q. Li J. Wang J. Dong W.. (2021). Widely targeted metabolomic analysis reveals dynamic changes in non-volatile and volatile metabolites during green tea processing. Food Chem. 363, 130131. doi: 10.1016/j.foodchem.2021.130131 34120048

[B41] Wang M. Huang H. Wang L. Yang H. He S. Liu F.. (2021). Herbal extract mixture modulates intestinal antioxidative capacity and microbiota in weaning piglets. Front. Microbiol. 12. doi: 10.3389/fmicb.2021.706758 PMC8357371 34394056

[B42] Xiao Y. Ren Q. Wu L. (2022). The pharmacokinetic property and pharmacological activity of acteoside: A review. Biomed. Pharmacother. 153, 113296. doi: 10.1016/j.biopha.2022.113296 35724511 PMC9212779

[B43] Yang W. J. Liu C. Gu Z. Y. Zhang X. Y. Cheng B. Mao Y.. (2014). Protective effects of acacetin isolated from Ziziphora clinopodioides Lam. (Xintahua) on neonatal rat cardiomyocytes. Chin. Med. 9, 28. doi: 10.1186/s13020-014-0028-3 25558275 PMC4272544

[B44] Yeh Y. Doan L. H. Huang Z. Chu L. Shi T. Lee Y.. (2022). Honeysuckle (*Lonicera japonica*) and huangqi (*Astragalus membranaceus*) suppress SARS-CoV-2 entry and COVID-19 related cytokine storm in *vitro* . Front. Pharmacol. 12. doi: 10.3389/fphar.2021.765553 PMC8990830 35401158

[B45] Yu X. (2013). *Preliminary research of anthocyanidins in lonicera japonica* var. *Chinensis and its formation mechanism* (Wuhan: Wuhan Polytechnic University).

[B46] Yuan Y. Yang J. Yu X. Huang L. Lin S. (2014). Anthocyanins from buds of *Lonicera japonica* Thunb. Var. *Chinensis* (wats.) Bak. Food Res. Int. 62, 812–818. doi: 10.1016/j.foodres.2014.03.026

[B47] Zhao X. Li H. Wang J. Guo Y. Liu B. Deng X.. (2016). Verbascoside alleviates pneumococcal pneumonia by reducing pneumolysin oligomers. Mol. Pharmacol. 89, 376–387. doi: 10.1124/mol.115.100610 26700563

[B48] Zhao Y. Jiang C. Lu J. Sun Y. Cui Y. (2023). Research progress of proanthocyanidins and anthocyanidins. Phytother. Res. 37, 2552–2577. doi: 10.1002/ptr.7850 37118988

[B49] Zhou Y. Tang R. C. (2018). Facile and eco-friendly fabrication of AgNPs coated silk for antibacterial and antioxidant textiles using honeysuckle extract. J. Photochem. Photobiol. B. 178, 463–471. doi: 10.1016/j.jphotobiol.2017.12.003 29223813

[B50] Zou S. Wu J. Shahid M. Q. He Y. Lin S. Liu Z.. (2020). Identification of key taste components in loquat using widely targeted metabolomics. Food Chem. 323, 126822. doi: 10.1016/j.foodchem.2020.126822 32334307

