# Supplementary material for: Unveiling the potential applications of buds of Lonicera japonica Thunb. var. chinensis (Wats.) Bak based on in vitro biological activities, bio-active components, and potential applications coupled to targeted metabolomics
Source: Front Plant Sci. 2024 Sep 26;15:1418957. doi: 10.3389/fpls.2024.1418957 (PMC11464324; doi:10.3389/fpls.2024.1418957)
Supplement: Supplementary file 1 [file DataSheet1.zip › Supplementary filesú¿revisedú⌐/Supplementary Figure.docx]

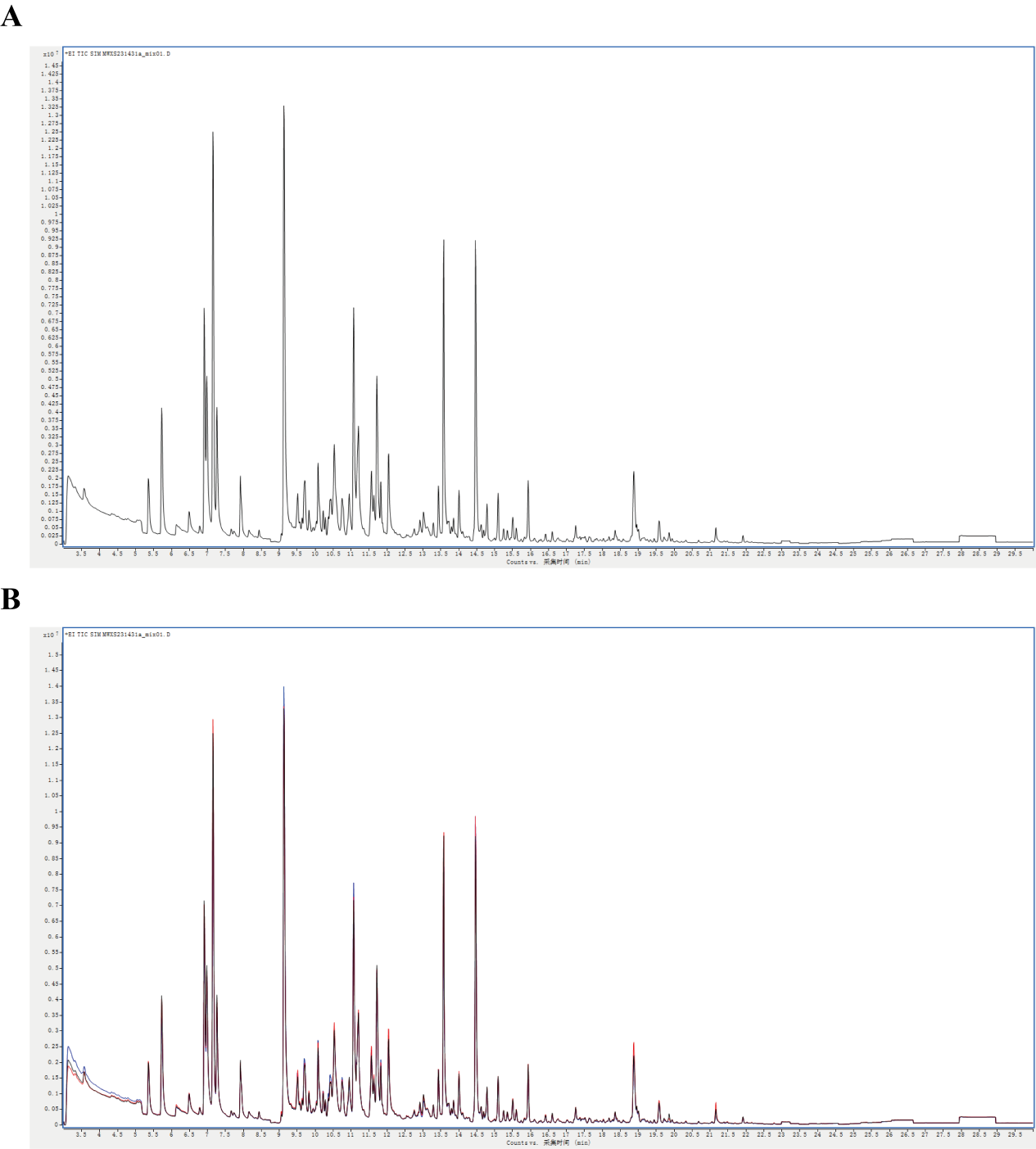


**Fig. S1** The single (A) and overlapping (B) TIC of QC samples by GC-MS.


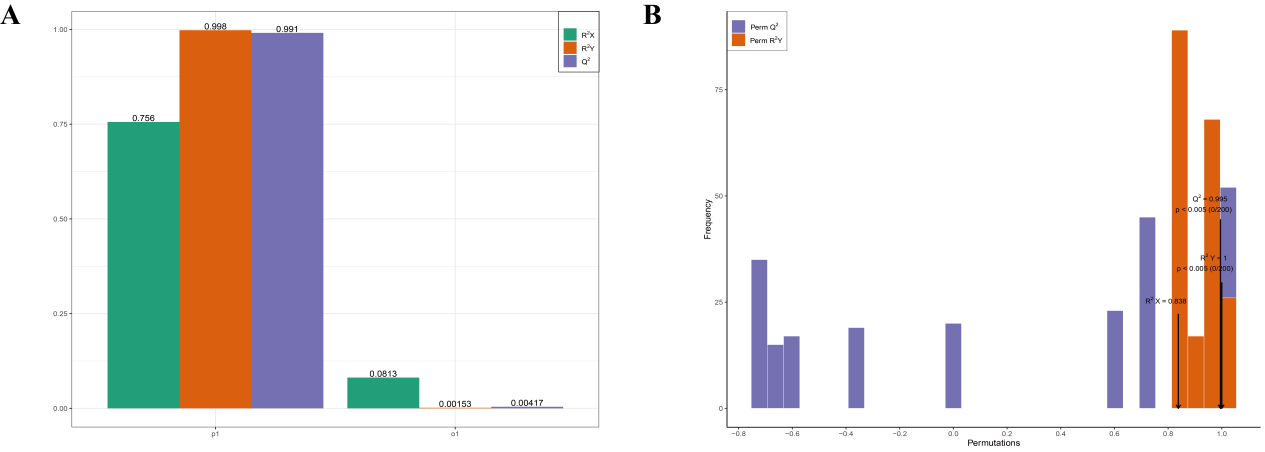


**Fig. S2** The OPLS-DA model validity (A) and permutation (B) in volatile metabolites of honeysuckle samples.


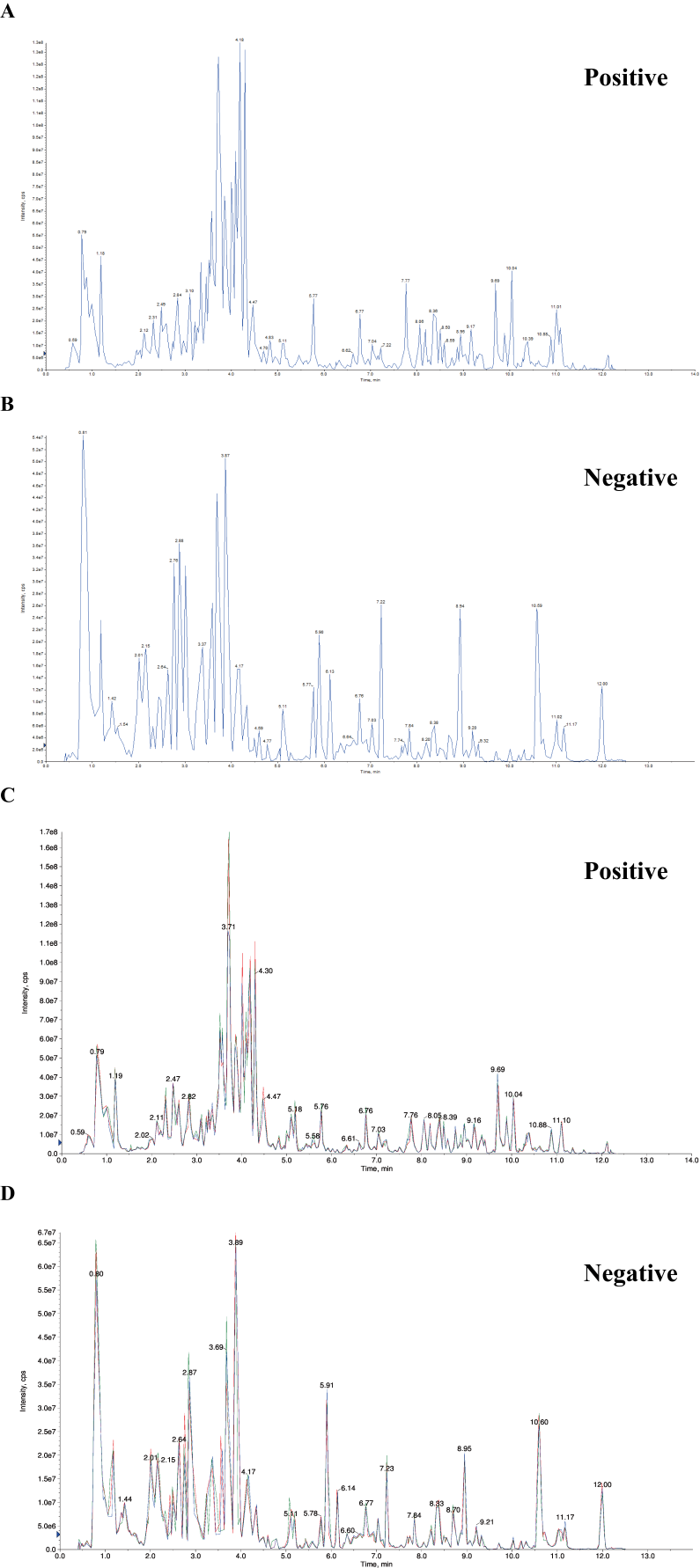


**Fig. S3** The single (A, B) and overlapping (C, D) TIC of QC samples in positive and negative by UPLC-MS/MS.


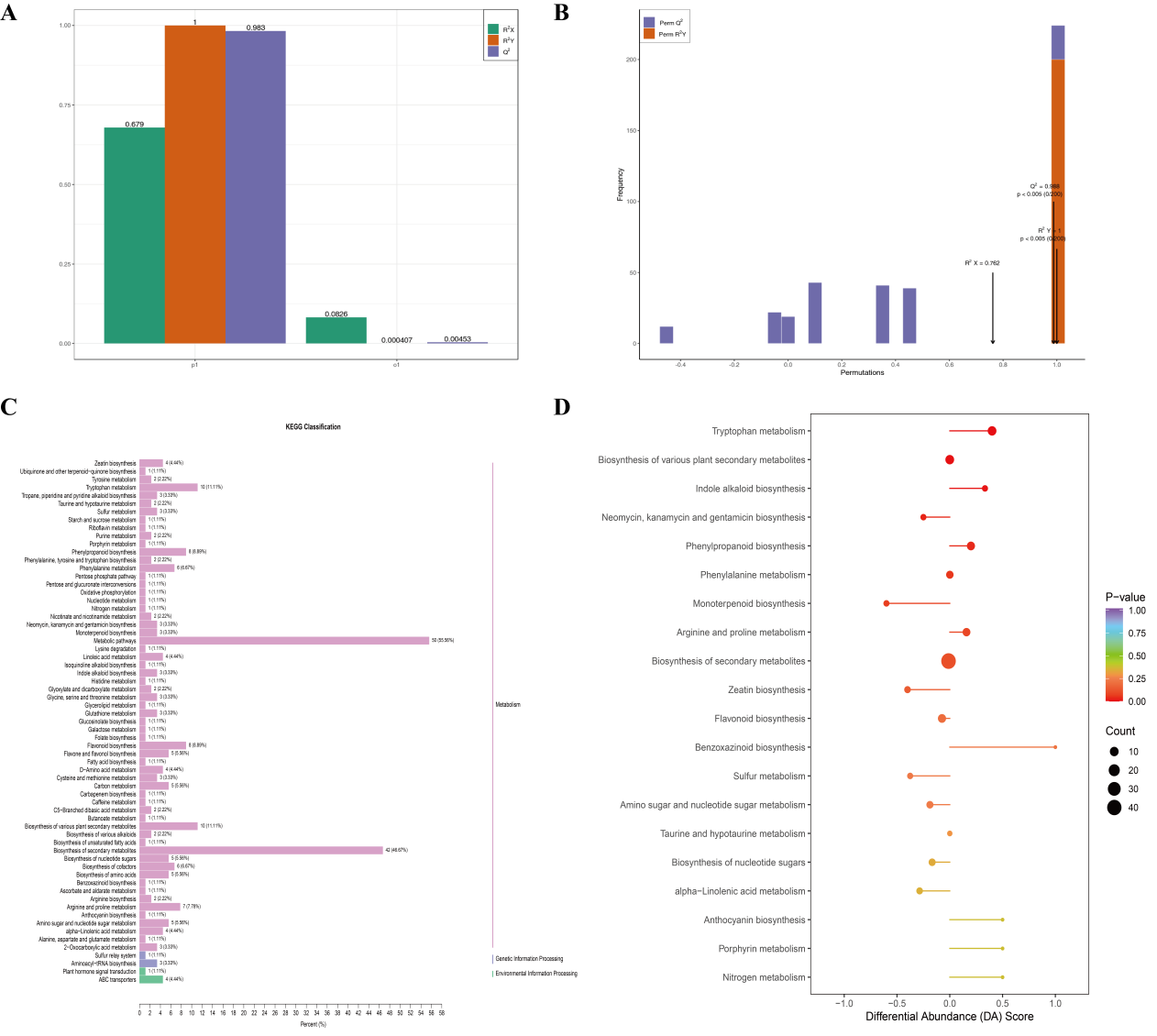


**Fig. S4** The validity (A) and permutation (B) of OPLS-DA model and metabolic pathways and differential abundance score of differential metabolites in non-volatile metabolites of honeysuckle samples.
