# Supplementary material for: Unveiling the potential applications of buds of Lonicera japonica Thunb. var. chinensis (Wats.) Bak based on in vitro biological activities, bio-active components, and potential applications coupled to targeted metabolomics
Source: Front Plant Sci. 2024 Sep 26;15:1418957. doi: 10.3389/fpls.2024.1418957 (PMC11464324; doi:10.3389/fpls.2024.1418957)
Supplement: Supplementary file 1 [file DataSheet1.zip › Supplementary filesú¿revisedú⌐/Supplementary Table.docx]

**Table S1**

Primer sequences involved in rt-qPCR.

| Primer | Primer sequence (5′ to 3′) | Product size (bp) |
| --- | --- | --- |
| TNF-α-F | CGGGCAGGTCTACTTTGGAG | 166 |
| TNF-α-R | ACCCTGAGCCATAATCCCCT |  |
| IL-1β-F | GCCTGTGTTTTCCTCCTTGC | 108 |
| IL-1β-R | TGCTGCCTAATGTCCCCTTG |  |
| IL-6-F | ACAAAGCCAGAGTCCTTCAGAG | 112 |
| IL-6-R | AGGAGAGCATTGGAAATTGGG |  |
| GAPDH-F | ACTCTTCCACCTTCGATGCC | 193 |
| GAPDH-R | TGGGATAGGGCCTCTCTTGC |  |

**Table S2**

The volatile differential metabolites between green and red honeysuckle samples.

| Compounds | Classification | Formula | CAS | Type |
| --- | --- | --- | --- | --- |
| (*Z*)-2-Butenoic acid, ethyl ester | Ester | C6H10O2 | 6776-19-8 | down |
| 2-Pentyl-furan | Heterocyclic compound | C9H14O | 3777-69-3 | down |
| 2-Methyl-1,3-dithiacyclopentane | Heterocyclic compound | C4H8S2 | 5616-51-3 | up |
| 2,6-Diethyl-Benzen amine | Amine | C10H15N | 579-66-8 | up |
| 2-Acetylbenzofuran | Heterocyclic compound | C10H8O2 | 1646-26-0 | up |
| cis-2-(2-Pentenyl)furan | Heterocyclic compound | C9H12O | 70424-13-4 | down |
| Benzamide | Amine | C7H7NO | 55-21-0 | up |
| 1-Methyl-4-(1-methylethylidene)-cyclohexene | Terpenoids | C10H16 | 586-62-9 | up |
| (*E*,*E*)-2,4-Decadienal | Aldehyde | C10H16O | 25152-84-5 | down |
| 5-Butyl-1,3-cyclohexadiene | Hydrocarbons | C10H16 | 30168-57-1 | up |
| (*Z*)-3,7-Dimethyl-1,3,6-octatriene | Terpenoids | C10H16 | 3338-55-4 | up |
| 4-Methyl-1-hexanol | Alcohol | C7H16O | 818-49-5 | down |
| 4,5-Dihydro-5,5-dimethyl-1H-pyrazole | Heterocyclic compound | C5H10N2 | 4320-85-8 | up |
| Eucalyptol | Terpenoids | C10H18O | 470-82-6 | down |
| 4-Methyl-1-(1-methylethenyl)-cyclohexene | Hydrocarbons | C10H16 | 586-67-4 | down |
| Paroxypropione | Ketone | C9H10O2 | 70-70-2 | up |
| Cubenene | Terpenoids | C15H24 | 29837-12-5 | up |
| 7-Methyl-8H-imidazo[1,2-*α*]pyrimidin-5-one | Terpenoids | C15H24 | 395070-76-5 | down |
| 6-Hexyltetrahydro-2H-pyran-2-one | Ester | C11H20O2 | 710-04-3 | down |
| 1,6-Dimethyl-4-(1-methylethyl)-naphthalene | Terpenoids | C15H18 | 483-78-3 | up |
| *D*-Limonene | Terpenoids | C10H16 | 5989-27-5 | up |
| 3,4-Dimethyl-1,5-cyclooctadiene | Hydrocarbons | C10H16 | 21284-05-9 | up |
| trans-Calamenene | Terpenoids | C15H22 | 73209-42-4 | up |
| Benzophenone | Ketone | C13H10O | 119-61-9 | down |
| 2-isopropyl-5-methyl-9-methylene-bicyclo[4.4.0]dec-1-ene | Terpenoids | C15H24 | 150320-52-8 | up |
| 2,6-Dimethyl-2,4,6-octatriene | Terpenoids | C10H16 | 673-84-7 | up |
| 2-Methyl-5-(1-methylethyl)-pyrazine | Heterocyclic compound | C8H12N2 | 13925-05-8 | up |
| 2-Ethyl-benzaldehyde | Aldehyde | C9H10O | 22927-13-5 | down |
| 2-Methyl-butanoic acid hexyl ester | Ester | C11H22O2 | 10032-15-2 | down |
| 2-Methyl-5-propan-2-ylcyclohexa-2,5-diene-1,4-dione | Ketone | C10H12O2 | 490-91-5 | up |
| 4-Isopropyl-6-methyl-1-methylene-1,2,3,4-tetrahydronaphthalene | Terpenoids | C15H20 | 50277-34-4 | up |
| (3*R*,3*αR*,7*S*,8*αS*)-3,6,8,8-Tetramethyl-4,7,8,8a-tetrahydro-1H-3a,7-methanoazulen-2(3H)-one | Terpenoids | C15H22O | 288249-25-2 | down |
| Benzyl Benzoate | Ester | C14H12O2 | 120-51-4 | down |
| 2,3,4,5-Tetrahydro-pyridine | Heterocyclic compound | C5H9N | 505-18-0 | down |
| (*E*,*E*)-2,4-Heptadien-1-ol | Alcohol | C7H12O | 33467-79-7 | down |
| 3-propyl-pyridine | Heterocyclic compound | C8H11N | 4673-31-8 | up |
| 1-Methyl-4-(1-methylethyl)-1,3-cyclohexadiene | Terpenoids | C10H16 | 99-86-5 | up |
| 2(1H)-Pyridinone | Heterocyclic compound | C5H5NO | 142-08-5 | down |
| 3,5-Diethyl-2-methyl-pyrazine | Heterocyclic compound | C9H14N2 | 18138-05-1 | up |
| 2-Methyl-naphthalene | Aromatics | C11H10 | 91-57-6 | down |
| (1*αR*,1*βS*,2*αS*,5*S*,5*αS*,7*αS*)-2,2,5,7*α*-Tetramethyldecahydrocyclopenta[2',3']cyclobuta[1',2':3,4]benzo[1,2-b]oxirene | Terpenoids | C15H24O | 104188-24-1 | down |
| Hexadecane | Hydrocarbons | C16H34 | 544-76-3 | down |
| 3-Nonen-5-one | Ketone | C9H16O | 82456-34-6 | down |
| 1-(furan-2-yl)-2-Methylpentan-1-one | Heterocyclic compound | C10H14O2 | 1248070-62-3 | down |
| 4-Propyl-phenol | Phenol | C9H12O | 645-56-7 | down |
| Phenylacetic acid propyl ester | Ester | C11H14O2 | 4606-15-9 | down |
| Copaene | Terpenoids | C15H24 | 3856-25-5 | up |
| 2,3-Dimethyl-quinoxaline | Heterocyclic compound | C10H10N2 | 2379-55-7 | up |
| Dodecanoic acid methyl ester | Ester | C13H26O2 | 111-82-0 | down |
| 1H-Pyrrole-2-carboxAldehyde | Heterocyclic compound | C5H5NO | 1003-29-8 | down |
| Hexanoic Acid | Acid | C6H12O2 | 142-62-1 | down |
| trans-*β*-Ocimene | Terpenoids | C10H16 | 3779-61-1 | up |
| Hexanoic acid 3-hexenyl ester | Ester | C12H22O2 | 84434-19-5 | down |
| 2-((3,3-Dimethyloxiran-2-yl)methyl)-3-methylfuran | Heterocyclic compound | C10H14O2 | 92356-06-4 | up |
| Naphthalene | Aromatics | C10H8 | 91-20-3 | down |
| Panaxene | Terpenoids | C15H24 | 871660-95-6 | down |
| 1-Phenyl-3-hexanone | Ketone | C12H16O | 29898-25-7 | up |
| (1*S*-cis)-1,2,3,5,6,8a-Hexahydro-4,7-dimethyl-1-(1-methylethyl)-naphthalene | Terpenoids | C15H24 | 483-76-1 | up |
| 2-Methoxy-dibenzofuran | Heterocyclic compound | C13H10O2 | 20357-70-4 | up |
| *β*-Myrcene | Terpenoids | C10H16 | 123-35-3 | up |
| Acetamide | Amine | C2H5NO | 60-35-5 | up |
| 2-Methyl-4,6-Octadiyn-3-one | Ketone | C9H10O | 29743-33-7 | up |
| Octanoic acid methyl ester | Ester | C9H18O2 | 111-11-5 | down |
| Nitro-benzene | Aromatics | C6H5NO2 | 98-95-3 | down |
| (*Z*)-6-Nonen-1-ol acetate | Ester | C11H20O2 | 76238-22-7 | down |
| Nonane | Hydrocarbons | C9H20 | 111-84-2 | up |
| 2,6-Dimethyl-pyrazine, | Heterocyclic compound | C6H8N2 | 108-50-9 | up |
| *O*-Cymene | Terpenoids | C10H14 | 527-84-4 | up |
| 2,6-Dimethyl-naphthalene | Aromatics | C12H12 | 581-42-0 | down |
| Acetic acid phenyl ester | Ester | C8H8O2 | 122-79-2 | down |
| 1,4-Dithiane | Amine | C4H8S2 | 505-29-3 | down |
| 2-ethenyl-1,1-dimethyl-3-methylene-cyclohexane | Hydrocarbons | C11H18 | 95452-08-7 | down |
| 1,1-dimethoxy-heptane | Hydrocarbons | C9H20O2 | 10032-05-0 | down |
| 3-Methyl-phenol | Phenol | C7H8O | 108-39-4 | down |
| Benzoic acid methyl ester | Ester | C8H8O2 | 93-58-3 | down |
| Benzenemethanethiol | Sulfur compounds | C7H8S | 100-53-8 | up |
| (1-methoxypropyl)-Benzene | Aromatics | C10H14O | 59588-12-4 | up |
| 1-Ethyl-3,5-dimethyl-benzene | Aromatics | C10H14 | 934-74-7 | up |
| Acetic acid 2-ethylhexyl ester | Ester | C10H20O2 | 103-09-3 | down |
| 1-Decen-3-one | Ketone | C10H18O | 56606-79-2 | down |
| Propanal dipropylhydrazone | Amine | C9H20N2 | 34687-35-9 | down |
| 1,2,3,4,4*α*,5,6,7-Octahydro-4a-methyl-naphthalene | Aromatics | C11H18 | 13943-77-6 | up |
| 1-Octen-1-ol acetate | Alcohol | C10H18O2 | 77149-68-9 | down |
| 1,2-Octanediol | Alcohol | C8H18O2 | 1117-86-8 | down |
| Nonanoic acid methyl ester | Ester | C10H20O2 | 1731-84-6 | down |
| (*Z*)-(3,3-dimethylcyclohexylidene)-Acetaldehyde | Aldehyde | C10H16O | 26532-24-1 | down |
| (*E*)-4-Hexen-1-ol | Alcohol | C6H12O | 928-92-7 | up |
| (*Z*)-4-Heptenal | Aldehyde | C7H12O | 6728-31-0 | up |
| Heptanal | Aldehyde | C7H14O | 111-71-7 | up |
| 3-Ethyl-3-methyl-diaziridine | Heterocyclic compound | C4H10N2 | 4901-75-1 | up |
| 3-(2H)-Pyridazinone | Heterocyclic compound | C4H4N2O | 504-30-3 | up |
| 2-Heptanone | Ketone | C7H14O | 110-43-0 | up |
| 1-Hepten-3-one | Ketone | C7H12O | 2918-13-0 | up |
| 2-Methyl-1-butanol acetate | Ester | C7H14O2 | 624-41-9 | up |
| 1H-Pyrazole | Heterocyclic compound | C3H4N2 | 288-13-1 | up |
| Ethyl-pyrazine | Heterocyclic compound | C6H8N2 | 13925-00-3 | down |
| 1-Methyl-cyclohexanol | Alcohol | C7H14O | 590-67-0 | up |
| Acetic acid pentyl ester | Ester | C7H14O2 | 628-63-7 | down |
| 1,4-Pentanediol | Alcohol | C5H12O2 | 626-95-9 | down |
| 2-Methyl-2-butenoic acid | Acid | C5H8O2 | 13201-46-2 | down |
| 3-Ethyl-3-methylheptane | Hydrocarbons | C10H22 | 17302-01-1 | down |
| 2,2,3,3-Tetramethyl-cyclopropanemethanol | Alcohol | C8H16O | 2415-96-5 | up |
| 1,2,4-Trimethyl-benzene | Aromatics | C9H12 | 95-63-6 | up |
| 4-Methylene-1-(1-methylethyl)-bicyclo[3.1.0]hex-2-ene | Terpenoids | C10H14 | 36262-09-6 | down |
| 4,6-Dimethyl-5-hepten-2-one | Ketone | C9H16O | 31162-48-8 | down |
| Dimethyl triSulfur compounds | Sulfur compounds | C2H6S3 | 3658-80-8 | down |
| Heptanoic acid methyl ester | Ester | C8H16O2 | 106-73-0 | down |
| Ethyl 1-methylethyl  2-methyl-3,4-dithiahexane | Sulfur compounds | C5H12S2 | 53966-36-2 | up |
| Isomaltol | Heterocyclic compound | C6H6O3 | 3420-59-5 | up |
| 2,6-Dimethyl-2-trans-6-octadiene | Terpenoids | C10H18 | 2609-23-6 | up |
| 4-Hexen-1-ol acetate | Alcohol | C8H14O2 | 72237-36-6 | down |
| 1,2-Hydrazinedicarboxaldehyde | Aldehyde | C2H4N2O2 | 628-36-4 | down |
| 1-(2-methyl-1-cyclopenten-1-yl)-Ethanone | Ketone | C8H12O | 3168-90-9 | down |
| Hexanoic acid methyl ester | Ester | C7H14O2 | 106-70-7 | down |
| (*E*)-3-Hexen-1-ol acetate | Ester | C8H14O2 | 3681-82-1 | up |
| nitroso-Benzene | Aromatics | C6H5NO | 586-96-9 | down |
| (*Z*)-3-Hexen-1-ol acetate | Ester | C8H14O2 | 3681-71-8 | down |
| 1-Methylene-4-(1-methylethenyl)-cyclohexane | Terpenoids | C10H16 | 499-97-8 | up |
| Indane | Aromatics | C9H10 | 496-11-7 | up |
| 3-Octen-2-one | Ketone | C8H14O | 1669-44-9 | down |
| Acetophenone | Ketone | C8H8O | 98-86-2 | down |
| 1,4-Diethyl-benzene | Aromatics | C10H14 | 105-05-5 | up |
| *β*-Phellandrene | Terpenoids | C10H16 | 555-10-2 | up |
| 2-Methyl-benzaldehyde | Aldehyde | C8H8O | 529-20-4 | down |
| 1-Octanol | Alcohol | C8H18O | 111-87-5 | down |
| (*E*,*E*)-3,5-Octadien-2-one | Ketone | C8H12O | 30086-02-3 | down |
| *γ*-Terpinene | Terpenoids | C10H16 | 99-85-4 | up |
| 2,5-FurandicarboxAldehyde | Aldehyde | C6H4O3 | 823-82-5 | down |
| 2,7-Dimethyl-3,6-bis(methylene)-1,7-octadiene | Hydrocarbons | C12H18 | 16714-60-6 | down |
| 2-Ethyl-3,5-dimethyl-pyrazine | Heterocyclic compound | C8H12N2 | 13925-07-0 | up |
| Hexanoic acid propyl ester | Ester | C9H18O2 | 626-77-7 | up |
| (S)-2-Methyl-6-methylene-7-octen-4-ol | Terpenoids | C10H18O | 35628-05-8 | up |
| (E)-2-Octen-1-ol | Alcohol | C8H16O | 18409-17-1 | down |
| (1*α*.,2*α*,5*α*)-2-Methyl-5-(1-methylethyl)-bicyclo[3.1.0]hexan-2-ol | Terpenoids | C10H18O | 17699-16-0 | down |
| 1-Methyl-4-(1-methylethenyl)-benzene | Aromatics | C10H12 | 1195-32-0 | up |
| 2,5,9-Trimethyl-decane | Hydrocarbons | C13H28 | 62108-22-9 | down |
| 4,6-Dimethyl-2,7-nonadien-5-one | Ketone | C11H18O | 74630-80-1 | down |
| (*E*,*E*)-2,6-Dimethyl-2,4,6-octatriene | Terpenoids | C10H16 | 3016-19-1 | up |
| (*Z*)-3-Methyl-4-undecene | Hydrocarbons | C12H24 | 74645-87-7 | down |
| 4-Aminopyridine | Heterocyclic compound | C5H6N2 | 504-24-5 | down |
| endo-Borneol | Terpenoids | C10H18O | 507-70-0 | down |
| 5-Methyl-2-(1-methylethenyl)-cyclohexanone | Terpenoids | C10H16O | 529-00-0 | up |
| Isopinocarveol | Terpenoids | C10H16O | 6712-79-4 | up |
| cis-1-Methyl-2-(1-methylethenyl)-cyclobutaneethanol | Alcohol | C10H18O | 30820-22-5 | up |
| 2-Methoxy-3-(2-methylpropyl)-pyrazine | Heterocyclic compound | C9H14N2O | 24683-00-9 | up |
| (*Z*)-Butanoic acid 4-hexenyl ester | Ester | C10H18O2 | 69727-41-9 | down |
| (*-*)-cis-Isopiperitenol | Terpenoids | C10H16O | 96555-02-1 | down |
| (+)-neodihydrocarveol | Terpenoids | C10H18O | 18675-33-7 | down |
| *α*-4-Dimethyl-3-cyclohexene-1-acetaldehyde | Aldehyde | C10H16O | 29548-14-9 | down |
| Anethole | Aromatics | C10H12O | 104-46-1 | down |
| dl-Camphoroquinone | Terpenoids | C10H14O2 | 10373-78-1 | down |
| 2-Acetyl-4-methyl-1,3-cyclopentanedione | Ketone | C8H10O3 | 4056-69-3 | down |
| (*S*)-4-(1-methylethenyl)-1-Cyclohexene-1-carboxaldehyde | Aldehyde | C10H14O | 18031-40-8 | down |
| (*E*)-5,9-Dimethyl-5,8-decadien-2-one | Ketone | C12H20O | 130876-99-2 | down |
| (4-methoxyphenyl)-Hydrazine | Aromatics | C7H10N2O | 3471-32-7 | down |
| 3-Hydroxy-hexanoic acid ethyl ester | Ester | C8H16O3 | 2305-25-1 | down |
| Butanoic acid phenylmethyl ester | Ester | C11H14O2 | 103-37-7 | up |
| (*Z*)-3-Methyl-2-(2-pentenyl)-2-cyclopenten-1-one | Ketone | C11H16O | 488-10-8 | up |
| Triacetin | Ester | C9H14O6 | 102-76-1 | up |
| Benzenepropanoic acid ethyl ester | Ester | C11H14O2 | 2021-28-5 | up |
| *α*-Terpinyl acetate | Ester | C12H20O2 | 80-26-2 | up |
| Hexanoic acid hexyl ester | Ester | C12H24O2 | 6378-65-0 | down |
| Biphenyl | Aromatics | C12H10 | 92-52-4 | down |
| (3*R*,6*R*)-2,2,6-Trimethyl-6-vinyltetrahydro-2H-pyran-3-ol | Heterocyclic compound | C10H18O2 | 14009-71-3 | up |
| 4-(2,6,6-trimethyl-2-cyclohexen-1-yl)-2-Butanone | Ketone | C13H22O | 31499-72-6 | up |
| 4-Hydroxy-benzenemethanol | Alcohol | C7H8O2 | 623-05-2 | up |
| cis-*α*-Bergamotene | Terpenoids | C15H24 | 18252-46-5 | down |
| trans-*β*-Ionone | Terpenoids | C13H20O | 79-77-6 | down |
| 1-Methyl-4-(1,2,2-trimethylcyclopentyl)cyclohexa-1,3-diene | Terpenoids | C15H24 | 29621-78-1 | up |
| 4-(1,5-dimethyl-1,4-hexadienyl)-1-Methyl-cyclohexene | Terpenoids | C15H24 | 17627-44-0 | up |
| 2-Naphthalenol | Phenol | C10H8O | 135-19-3 | up |
| Citronellyl butyrate | Ester | C14H26O2 | 141-16-2 | down |
| (*R*)-2,4*α*,5,6,7,8-Hexahydro-3,5,5,9-tetramethyl-1H-benzocycloheptene, | Terpenoids | C15H24 | 1461-03-6 | up |
| Benzoic acid hexyl ester | Ester | C13H18O2 | 6789-88-4 | down |
| (*E*)-2-Methyl-butanoic acid 3,7-dimethyl-2,6-octadienyl ester | Ester | C15H26O2 | 68705-63-5 | down |
| 1,8-Dimethyl-8,9-epoxy-4-isopropyl-spiro[4.5]decan-7-one | Terpenoids | C15H24O2 | 61050-91-7 | down |
| Ibuprofen | Acid | C13H18O2 | 15687-27-1 | down |
| 1,1'-(1,3-propanediyl)bis-benzene | Aromatics | C15H16 | 1081-75-0 | down |
